# ACT001 attenuates microglia-mediated neuroinflammation after traumatic brain injury via inhibiting AKT/NFκB/NLRP3 pathway

**DOI:** 10.1186/s12964-022-00862-y

**Published:** 2022-04-23

**Authors:** Lin Cai, Qiuyuan Gong, Lin Qi, Tongtong Xu, Qian Suo, Xiang Li, Wei Wang, Yao Jing, Dianxu Yang, Zhiming Xu, Fang Yuan, Yaohui Tang, Guoyuan Yang, Jun Ding, Hao Chen, Hengli Tian

**Affiliations:** 1 Department of Neurosurgery, Shanghai Jiao Tong University Affiliated Sixth People’s Hospital, Shanghai, 200233 China; 2 Med-X Research Institute and School of Biomedical Engineering, Shanghai Jiao Tong University, Shanghai, 200033 China; 3 Department of Osteoporosis and Bone Disease, Shanghai Jiao Tong University Affiliated Sixth People’s Hospital, Shanghai, 200233 China

**Keywords:** ACT001, Traumatic brain injury, Microglia cells, Neuroinflammation, AKT, NFκB, NLRP3 inflammasome

## Abstract

**Background:**

Microglia-mediated neuroinflammatory response following traumatic brain injury (TBI) is considered as a vital secondary injury factor, which drives trauma-induced neurodegeneration and is lack of efficient treatment. ACT001, a sesquiterpene lactone derivative, is reportedly involved in alleviation of inflammatory response. However, little is known regarding its function in regulating innate immune response of central nervous system (CNS) after TBI. This study aimed to investigate the role and underlying mechanism of ACT001 in TBI.

**Methods:**

Controlled cortical impact (CCI) models were used to establish model of TBI. Cresyl violet staining, evans blue extravasation, neurobehavioral function assessments, immunofluorescence and transmission electron microscopy were used to evaluate therapeutic effects of ACT001 in vivo. Microglial depletion was induced by administering mice with colony stimulating factor 1 receptor (CSF1R) inhibitor, PLX5622. Cell-cell interaction models were established as co-culture system to simulate TBI conditions in vitro. Cytotoxic effect of ACT001 on cell viability was assessed by cell counting kit-8 and activation of microglia cells were induced by Lipopolysaccharides (LPS). Pro-inflammatory cytokines expression was determined by Real-time PCR and nitric oxide production. Apoptotic cells were detected by TUNEL and flow cytometry assays. Tube formation was performed to evaluate cellular angiogenic ability. ELISA and western blot experiments were used to determine proteins expression. Pull-down assay was used to analyze proteins that bound ACT001.

**Results:**

ACT001 relieved the extent of blood-brain barrier integrity damage and alleviated motor function deficits after TBI via reducing trauma-induced activation of microglia cells. Delayed depletion of microglia with PLX5622 hindered therapeutic effect of ACT001. Furthermore, ACT001 alleviated LPS-induced activation in mouse and rat primary microglia cells. Besides, ACT001 was effective in suppressing LPS-induced pro-inflammatory cytokines production in BV2 cells, resulting in reduction of neuronal apoptosis in HT22 cells and improvement of tube formation in bEnd.3 cells. Mechanism by which ACT001 functioned was related to AKT/NFκB/NLRP3 pathway. ACT001 restrained NFκB nuclear translocation in microglia cells through inhibiting AKT phosphorylation, resulting in decrease of NLRP3 inflammasome activation, and finally down-regulated microglial neuroinflammatory response.

**Conclusions:**

Our study indicated that ACT001 played critical role in microglia-mediated neuroinflammatory response and might be a novel potential chemotherapeutic drug for TBI.

**Video Abstract**

**Supplementary Information:**

The online version contains supplementary material available at 10.1186/s12964-022-00862-y.

## Background

Traumatic brain injury (TBI), a disease with structural or physiological cerebral dysfunction caused by external force strike [1], is one of the leading causes of death and disability worldwide [2]. It was estimated that approximately 5.3 million people in the USA and over 7.7 million people in Europe were suffering from the undesirable consequences of TBI [3], causing a huge burden to societies and families.

The pathology of TBI is heterogeneous and complex, with TBI commonly categorized into primary and secondary injuries. Primary injury is the result of mechanical forces acting at the moment of injury. The type and severity of injury depend upon the nature of initiating force, as well as the site and direction [4]. Following the mechanical insult [5], a cascade of cellular and biochemical changes, including inflammation, oxidative stress, mitochondrial dysfunction and apoptosis, initiate within minutes, leading to secondary injury, which can even last from months to years. Recently, mounting evidences from human and animal studies [6, 7] have demonstrated that sustained and excessive secondary injury could hinder the neuroprotection and neurorepair, leading to delayed and limited neurological function recovery after TBI. Among these pathological events, neuroinflammation has been recognized as a major pathological process [8, 9]. It was characterized by the release of pro-inflammatory cytokines and activation of innate immune response in CNS [10–12]. Although pre-clinical studies have shown that anti-inflammatory agents exhibited therapeutic effects on improving prognosis of animal TBI models, reports from clinical trials have been less promising [13, 14]. Therefore, new therapeutic drugs, aiming to restore the balance between pro-inflammatory and anti-inflammatory effect, are inevitably needed to improve the outcome after TBI.

Microglia, the major resident intrinsic immune cells of CNS, are known as main mediators of neuroinflammatory response to CNS injuries [11, 15, 16]. After TBI, the resident microglia cells are rapidly activated, mobilize to the damaged area to clear debris and induce a multitude of inflammatory cascades [10]. As such, microglial activation acts as a critical innate immune defense that is required to protect the brain from injury [17]. However, uncontrolled and excessive activation is detrimental and can even exacerbate neurodegenerative processes and neurological dysfunction [18]. Reportedly, dysregulated microglial activation can be sustained up to 17 years in TBI patients [19] and underlie the persistent neuropathological changes, such as delayed blood-brain barrier (BBB) repairment and neuronal apoptosis [10, 20, 21]. Multiple signaling pathways are implicated in modulating the activation of microglia after TBI, nevertheless, NFκB is still recognized as predominant transcription factor in regulating pro-inflammatory mediators [22–24]. In the “resting” state of microglia cell, NFκB is located in cytoplasm as a dimer consisting of p50 and p65 subunits. In response to TBI stimuli, the inhibitor of NFκB (IκB) is phosphorylated and degraded to release NFκB dimer, which is activated and translocated into nucleus, resulting in the transcription of pro-inflammatory factors, such as NLRP3 (NLR Family Pyrin Domain Containing 3) inflammasome activation. Thus, inhibiting the activation of NFκB is widely considered as an overwhelming therapeutic strategy for attenuating trauma-induced neuroinflammation.

ACT001 (also known as dimethylamino-micheliolide, i.e., DMAMCL) is certified as an orphan drug by the Food and Drug Administration (FDA) in the United States [25, 26]. It is derived from micheliolide (MCL), a natural product of sesquiterpene lactones (SLs) originally isolated from *Michelia compressa* and *Michelia champaca* [27–29]. SLs has a long history of use around the world for preventing migraine headaches, treating rheumatoid arthritis and anti-infection, actions that are attributable to its anti-inflammatory activity [30, 31]. Several studies have demonstrated that SLs was a potent inhibitor of NFκB activation and could inhibit the expression of pro-inflammatory cytokines in vivo and in vitro [32, 33]. However, the chemical properties of SLs are instable and can decompose easily in both acidic and basic conditions [34], which severely restrict their clinical application. DMAMCL is the dimethylamino Michael adduct of MCL and ACT001, a fumarate salt form of DMAMCL typically used in vivo [35], displays higher plasma stability, more sustained release and superior efficacy, which increases its oral bioavailability and enhances its therapeutic potential [36]. Besides, ACT001 can cross the BBB, a formidable obstacle for many drugs to exert therapeutic effect, and effectively inhibit glioblastoma multiforme (GBM) growth with low systemic and local toxicity [26, 37]. In all, these results indicate that ACT001 has great pharmacokinetic properties as a potential therapy for intracranial diseases [36–39]. However, the role of ACT001 in the treatment of TBI-induced neuroinflammation remains largely unclear.

In the present study, we aimed to investigate the anti-inflammatory effects and underlying mechanisms of ACT001 in TBI and found that ACT001 was effective in attenuating microglia-mediated neuroinflammation in vitro and in vivo via restraining NFκB nuclear translocation through inhibiting the phosphorylation of AKT. These novel findings suggest that ACT001 is a promising chemotherapeutic drug that is highly effective against microglia-mediated neuroinflammation after TBI.

## Materials and methods

### Cell culture and reagents

The murine microglia cell line, BV2 cells, were provided by the China Center for Type Culture Collection (CCTCC, Wuhan, Hubei, China), the murine hippocampal neuronal cell line, HT22 cells, and the murine brain endothelial cell line, bEnd.3 cells, were purchased from American Type Culture Collection (ATCC, Manassas, VA, USA). All the cells were maintained in complete Dulbecco’s modified Eagle medium (DMEM) supplemented with 10% heat-inactivated fetal bovine serum (FBS) and 100 U/ml penicillin/streptomycin, and incubated in a humidified incubator at 37 °C in 5% (v/v) CO_2_. ACT001 was generously provided by Accendatech Co., Ltd. (Tianjin, China). The details of reagents used in this study were shown in Additional file 1: Table S1.

### Animals

Primary microglia cells were isolated from the cortexes of postnatal day 1 to day 3 wild-type mice and Sprague-Dawley rats. TBI models were performed using adult male C57BL/6 mice (8–12 weeks old, 20–25 g). All animals were purchased from Shanghai SLAC Laboratory Animal Corp., (Shanghai, China). All operational procedures were performed in accordance with the guidelines of the Institutional Animal Care and Use Committee of Shanghai Jiao Tong University, Shanghai, China and reported according to the ARRIVE guidelines. Every effort has been made to minimize animal suffering.

### Primary microglia cells culture

Primary microglia cells culture was performed as previously described [40, 41]. Briefly, cortexes were removed aseptically from the skulls and meninges were excised carefully under a dissecting microscope. Primary glial cells were obtained from the cerebral cortices, which were earlier digested by 0.25% Trypsin/EDTA at 37 °C for 20 min and seeded into 0.01% poly-ℒ-lysine-coated (PLL) 75cm^2^ culture flasks. The cultures were maintained for 2 weeks in complete DMEM. Media was replaced one day after preparation and subsequently every 2–3 days. After mixed glial cultures were completely confluent, microglia cells were separated by shaking at 180 rpm on a gyratory shaker (Lab Companion SI-600, JeioTech Co. Ltd, Korea) for 30 min at 37  °C and then reseeded in culture dishes for subsequent research. The percentage of the primary microglia cells was confirmed by Iba1 staining with over 97% purity.

### TBI models

The CCI model of TBI is an open-head model of mechanical contusion injury to cortex. The impact parameters can be modified to produce CCI injuries that range from mild to severe [42]. After anesthetization with xylazine (10 mg/kg) and ketamine (75 mg/kg), the mice were secured in a stereotaxic frame (Stoelting, Wood Dale, IL, USA) with a hot pack placed under the body to maintain core body temperature at 37.0 ± 0.5 °C. The surgical area was disinfected with 75% alcohol and then a mid-line incision (1.5–2 cm) was made on the scalp. After that, a 4-mm-diameter craniotomy was performed using a portable drill over the right parietal cortex between *bregma* and *lambda*, 1 mm lateral to the midline. The dura mater was kept intact over the cortex and mice were excluded if dural integrity was breached. A contusion injury was performed perpendicular to brain surface by using a CCI device (PinPoint Precision Cortical Impactor PCI3000; Hatteras Instruments Inc., USA) with a rounded steel impactor probe (3-mm-diameter). The impact velocity was 1.5 m/s, the impact duration was 100 ms and the impact depth was 1.5 mm. After injury, the bone flap was sealed with sterile bone wax and the scalp was sutured with interrupted 6–0 silk sutures. All animals were placed in 37 °C heated cages until completely ambulatory. Sham animals underwent the same procedure as TBI group excepting for CCI.

### Drug administration

For in vivo studies, ACT001 was dissolved in 0.1 mmol phosphate buffered saline (PBS) to reach final concentrations of 20 mg/ml. PBS or ACT001 was administered daily by oral gavage (100 mg/kg) from the day of CCI surgery until 7 days after the surgery. CSF1R inhibitor, PLX5622, was provided by Plexxikon Inc. (Berkeley, CA) and formulated in AIN-76A standard rodent chow by Research Diets Inc. (New Brunswick, NJ) at a concentration of 1200 parts per million [ppm]. The specialized diet was stored in 4 °C. All mice had free access to PLX5622 diet to deplete microglia cells or AIN-76A chow as vehicle control. After being raised for 2 weeks, mice were subjected to CCI injury for further research.

LPS is the principal component of outer membrane of Gram-negative bacteria, which can significantly induce the activation of microglia cells and improve pro-inflammatory cytokine expression in a dose-dependent manner in vivo [43, 44]. In order to further evaluate whether the inhibitory effect of ACT001 on microglial activation could still work effectively with increased concentrations of LPS treatment in vitro, the concentrations of 100 ng/mL and 500 ng/mL were selected in this study as reported previously [45–48]. ACT001 was dissolved in cells culture medium at different concentrations (from 1 μM to 500 μM). After stimulating cells with LPS, ACT001 were added immediately.

### Brain Tissue preparation

Mice were anesthetized and transcardially perfused with PBS and 4% paraformaldehyde (PFA) at indicated time post-injury. Brain tissues post-fixed overnight in 4% PFA were dehydrated in 30% sucrose until they sank to the bottom of the liquid and then stored at −80 °C. Subsequently, 30 μm coronal sections from *bregma* −1.0 to −3.0 mm were collected in 24-well plates filled with PBS plus 0.05% sodium azide and stored at 4 °C until use.

### Lesion volume assessment

To measure the lesion volume in ipsilateral cortex after TBI, cresyl violet stained sections were digitized and analyzed by using ImageJ (National Institutes of Health, Bethesda, MD, USA). The total volume was computed by outlining the injured brain areas and multiplying with the inter-slice distance. Hemispheric tissue loss was calculated by the formula: Lesion Volume = [(contralateral hemispheric volume − ipsilateral hemispheric volume)/(contralateral hemispheric volume) × 100%].

### Measurement of evans blue (EB) extravasation

Alterations in microvascular permeability after TBI was evaluated by measuring the extravasation of EB. EB dye (0.2 ml/100 g) was injected through the femoral vein and circulated for 2 h. Then, the mice were anesthetized and sacrificed by cardiac perfusion. Subsequently, the brain was collected and divided into two hemispheres. Each sample was weighed immediately and homogenized with 1 mL of 50% trichloroacetic acid solution, then centrifuged at 12,000 × *g* for 20 min. The supernatant was transferred to mix with triple volumes of ethanol and measured with a spectrophotometer at a wavelength of 610 nm (BioTek, Winooski, VT, USA).

### Neurobehavioral function assessments

Neurobehavioral assessments were recorded at 1, 3, 5, 7, and 14 days after TBI by an investigator blinded to experimental design using the modified Neurological Severity Score (mNSS), Grid-Walking Test, Rotarod Test (Rotor-Rod) and Hanging Wire Test. The detailed criterias were shown in Additional file 2: Table S2.

### Immunofluorescence staining

4% PFA fixed cells or brain sections with antigen retrieval were treated with 100% methanol (chilled at −20 °C) for 10 min and then incubated with 1% bovine serum albumin (BSA) for 1 h. Next, cells or brain sections were incubated overnight with primary antibodies at 4 °C followed by fluorescent conjugated secondary antibodies for 1 h at 37 °C, after which they were stained with DAPI solution. Staining was visualized on an LSM710 laser scanning confocal fluorescence microscope with a 363-oil immersion lens (Carl Zeiss, Oberkochen, Germany), and images were obtained. The indicated antibodies were listed in Additional file 1: Table S1.

For positive staining quantification analysis, microglia cells, neurons and cerebral microvessels were imaged based on Iba1^+^, NeuN^+^ and CD31^+^ immunostaining, respectively. The numbers of positive staining cells with a signal-to-noise ratio (S/N) ≥ 10.0 were quantified to distinguish the positive fluorescence intensity (The Signal) from the cellular autofluorescence (The Noise). For each section, two or three fields were imaged in the defined space. Subsequent images were processed and quantified while the investigators were blind to the experimental conditions. The calculation methods have been described in detail as reported previously [49, 50].

### Terminal dexynucleotidyl transferase mediated dUTP nick end labeling (TUNEL) assay

Apoptotic cells were detected by TUNEL assay using One Step TUNEL Apoptosis Assay kit, which was performed following manufacturer's instruction. Briefly, the samples were subjected to react with the TUNEL mixture solution for 1 h at 37 °C, and then stained with DAPI. Apoptotic cells were observed and recorded under a confocal fluorescence microscope. The apoptosis rate was calculated by the formula: apoptotic cells/all cells in a field × 100%.

### Transmission electron microscopy (TEM)

Samples were processed in the Electron Microscopy Department at Shanghai Jiao Tong University. Cell pellets were fixed with 2.5% glutaraldehyde overnight in PBS and post-fixed with 1% osmium tetroxide (pH 7.4) for 2 h at room temperature. The pellets were then dehydrated in a graded ethanol series and infiltrated with Spurr’s resin to embed the tissues. Samples were then polymerized for 48 h at 60 °C, cut into 60-nm-thick sections on an LKB-I microtome (LKB, Sweden), positioned on 200-mesh grids and stained with uranyl acetate and lead citrate. TEM was performed on a PHILIPS CM120 transmission electron microscope at an accelerating voltage of 120 kV. Images were acquired with a Gatan-type UltraScan 4000SP CCD camera (Pleasanton, CA) connected to the microscope.

### Cell viability assays

Cell viability was measured by Cell Counting kit-8 (CCK-8) assay according to manufacturer’s protocol. Cells (8000–10,000 per well) were plated in 96-well plates and cultured overnight. Then different concentrations of LPS, ACT001 or PBS alone as control were treated with cells. At indicated time after treatment, 10 μL CCK-8 solution was added to 90 μL of culture medium. The cells were subsequently incubated for 3 h and the absorbance was read at 450 nm using a spectrophotometer (TECAN, Switzerland).

### Real-time PCR

Total RNA was extracted from BV2 cells by using TRIzol™ reagent, according to manufacturer's instruction. RNA concentrations were examined by the spectrophotometer (NanoDrop 1000, Thermo). First-strand cDNAs were synthesized using cDNA Synthesis SuperMix kit. Each cDNA (2 μL) was amplified using Hieff qPCR SYBR Green Master Mix (final volume, 20 mL) and analysed on the Applied Biosystems 7900 Real-time PCR Detection System. Thermal cycling conditions were performed as follows: melting step (95 °C for 30 s), annealing step (40 cycles of 95 °C for 10 s), and elongation (60 °C for 30 s). RNA primers were listed in Additional file 3: Table S3.

### Measurement of NO Production

Nitric oxide (NO) production was determined indirectly through the measurement of nitrite released into microglial culture supernatants using Total Nitric Oxide Assay kit. 50 μL supernatants were mixed with equal volume of Griess reagent, incubated for 30 min at room temperature and the absorbance was measured at 540 nm using a microplate reader (Tecan, Hombrechtikon, Switzerland). Results were expressed as percentage of control and sodium nitrite reference curve was prepared for each determination.

### The cell–cell interaction models

To establish an in vitro co-culture system model, BV2 cells were indirectly co-cultured with HT22 or bEnd.3 cells using a Transwell culture system (0.4 μm pore size, Corning, NY, USA). 10^5^ BV2 cells were cultured in upper chambers and pre-treated with LPS or PBS alone as control for 24 h, while 3 × 10^5^ HT22 or bEnd.3 cells were added into the bottom of 24-well plates and allowed to attach overnight. After that, the upper chambers were switched into 24-well plates and co-cultured in ACT001-added complete DMEM for 24–48 h. Then, the culture supernatants were centrifuged at 2000 × g for 10 min to remove cell debris and used immediately or frozen at −80 °C after allocation. The cells in lower chambers were harvested for further research.

### Cell death analysis by flow cytometry

To determine the cell death of HT22 cells induced by BV2-secreted pro-inflammatory cytokines, PE Annexin V Apoptosis Detection Kit I was used according to manufacturer's instruction. A total of ≥ 10^4^ cells were resuspended in binding buffer and incubated with 5 µL of Annexin V-FITC and 5 µL of PI for 15 min in dark. The proportions of cells in early apoptosis and late apoptosis were reported as percentage of Annexin V^+^/PI^−^- and Annexin V^+^/PI^+^-labeled cells, respectively. The stained cells were analyzed directly by flow cytometry using a FACSCalibur with the Cell Quest program (BD Biosciences, USA) for data analysis.

### Tube formation assay

To evaluate the angiogenic ability of co-cultured bEnd.3 cells, tube formation was performed as described before [51]. Briefly, Chilled liquid growth factor reduced Matrigel Matrix was prepared in serum-free cold DMEM at a dilution of 1:1 and dispensed into 24-well plates (200 μL/well). After being incubated at 37 °C for 1 h, bEnd.3 cells were seeded in each Matrigel-coated well and co-cultured with pre-treated BV2 cells for 24 h. Tube formation was observed and captured with a phase contrast microscope.

### Enzyme-linked immunosorbent assay (ELISA)

VEGF expression was measured using a commercially available ELISA kit according to the manufacturer’s instruction. The minimal detectable dose of mouse VEGF concentration was 3.0 pg/mL with inter-assay and intra-assay coefficients of variation less than 10%.

### Immunoblotting

Cell lysates were extracted with cell lysis buffer and the protein concentrations in lysates were quantified using an Enhanced BCA Protein Assay kit. Protein samples (30–50 µg) were separated by SDS-PAGE and then transferred to PVDF membranes. The membranes were immunoblotted with primary antibodies followed by HRP-linked secondary antibodies. Immunoreactive proteins were visualized using ECL western blot detection kit and images were developed using a Bio-Rad system (Bio-Rad, USA). The indicated antibodies were listed in Additional file 1: Table S1.

### Pull-down of ACT001-biotin bound proteins

ACT001-biotin-bound proteins were isolated as described previously [38, 52]. We constructed ACT001-biotin probe as positive group and ACT001-S-biotin probe as negative group. Briefly, cells were lysed and centrifuged, and the supernatant (1.5 mg/mL) was collected and equally divided into three samples, which were used in the protein pull-down assay. One supernatant sample was used as input group. The other two samples were either incubated with 100 μM (active) ACT001-biotin or ACT001-S-biotin (inactive ACT001-biotin) in RIPA buffer overnight at 4 °C. Then, the samples were incubated with streptavidin beads, separated by SDS-PAGE, and visualized by silver staining and western blot assays.

### Statistical analysis

All statistical analyses were carried out using SPSS 16.0 statistical software package (SPSS Inc., Chicago, IL, USA). Continuous variables were expressed as means ± SE. In each experiment, all conditions were evaluated at least in triplicate. Statistical significance between two measurements was determined by two-tailed unpaired Student’s *t* test, and among groups of three or more, it was determined by one-way analysis of variance (*ANOVA*). *P* values < 0.05 were considered statistically significant.

## Results

### ACT001 relieved the extent of neurological impairment and alleviated motor function deficits in mice after TBI

**Fig. 1:**
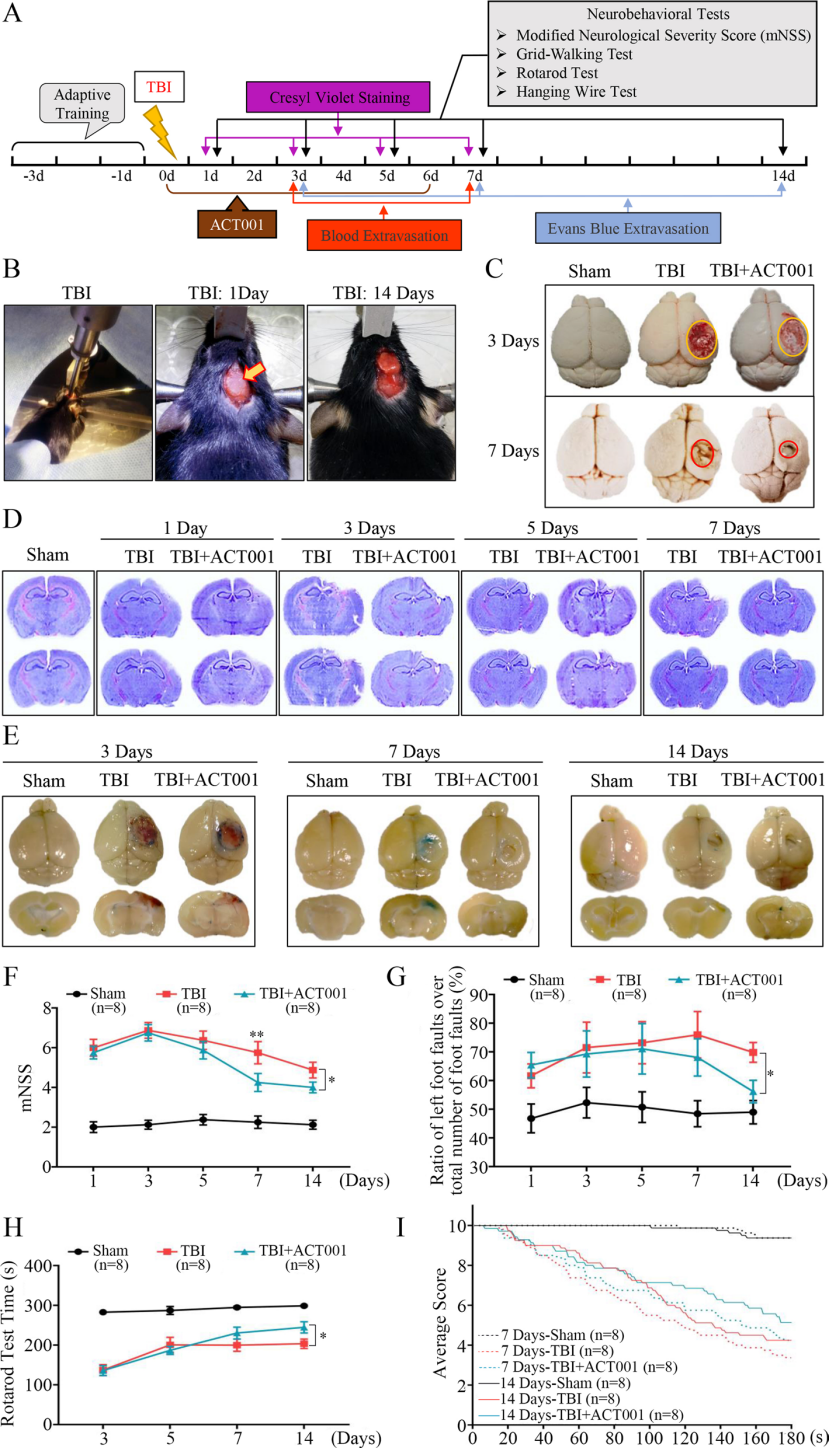
ACT001 attenuated neurological impairment and promoted neurobehavioral recovery in mice CCI models. **a** Overview of experimental timeline for in vivo studies and neurobehavioral tests. **b** Surgical process illustration of CCI models. After contusion injury (left), the bone flap was sealed with sterile bone wax (middle), indicated by orange arrow. The peri-contusional tissue region at 14 days post-insult was shown on the right panel. **c** Representative photographs of whole brains in Sham, TBI and TBI + ACT001 groups at 3 and 7 days post-insult. The circles represented the approximate area of lesion cortex. **d** Representative cresyl violet-stained brain sections at indicated time points post-insult. n = 6/group. **e** Representative images of EB extravasation in three groups at indicated time points post-insult (blue areas indicated the dye extravasation). n = 6/group. **f-i** Line graphs showed four neurobehavioral function assessments at indicated time points including mNNS scores (**f**), Grid-Walking test (**g**), Rotarod test (**h**) and Hanging Wire test (**i**). n = 8/group. Data were presented as mean ± SEMs, *P < 0.05, **P < 0.01, versus TBI group

Mounting evidences have shown that ACT001 was effective in neurological disease treatment [28, 35, 36, 53]. However, little is known regarding its role in TBI. Therefore, we constructed CCI models to examine the therapeutic effect of ACT001 on TBI. The experimental timeline was shown in Fig. 1a, all mice used to assess neurobehavioral outcomes would undergo adaptive training for 3 days before TBI. After model establishment, the bone flap was sealed with sterile bone wax to maintain structural integrity (Fig. 1b), and mice were randomly divided into Sham group, TBI group and TBI + ACT001 group for subsequent experiments. Firstly, extravasated blood present in ipsilateral cortex was quantified at 3 and 7 days post-TBI. The amount in ACT001 + TBI group was significantly less than that in TBI group at 7 days, and there was no significant difference at 3 days (Fig. 1c). Additionally, consecutive brain contusion sections stained with cresyl violet were used to assess gross pathological changes between TBI and ACT001 + TBI groups. As demonstrated, TBI caused tissue defect in ipsilateral cortex over time, and ACT001 administration could partly relieve the extent of lesion volume (Fig.1 d). Compared with TBI group, lesion volume in ACT001 + TBI groups decreased by 5.36 ± 1.41% at 7 days after TBI (*P* < 0.001; Additional file 4: Fig. S1A). Then, EB extravasation assay was performed to assess protective function of ACT001 on BBB disruption (Fig. 1e). Statistical analyses revealed that ACT001 + TBI group exhibited lower EB leakage level in ipsilateral cortex compared with TBI group at 7 days (*P* < 0.001; Additional file 4: Fig. S1B) and no significant differences at 3 or 14 days. Next, we evaluated neurobehavioral function recovery in mice after TBI. As compared with sham group, TBI mice showed much severer neurobehavioral deficiency (Fig. 1f–i). Interestingly, ACT001 administration exhibited a certain extent improvements in mNSS scores (*P* < 0.05, Fig. 1f), Grid-Walking test (*P* < 0.05, Fig. 1g), Rotarod test (*P* < 0.05, Fig. 1h) and Hanging Wire test (*P* < 0.001, Fig. 1i) at 14 days post-TBI.

**Fig. 2:**
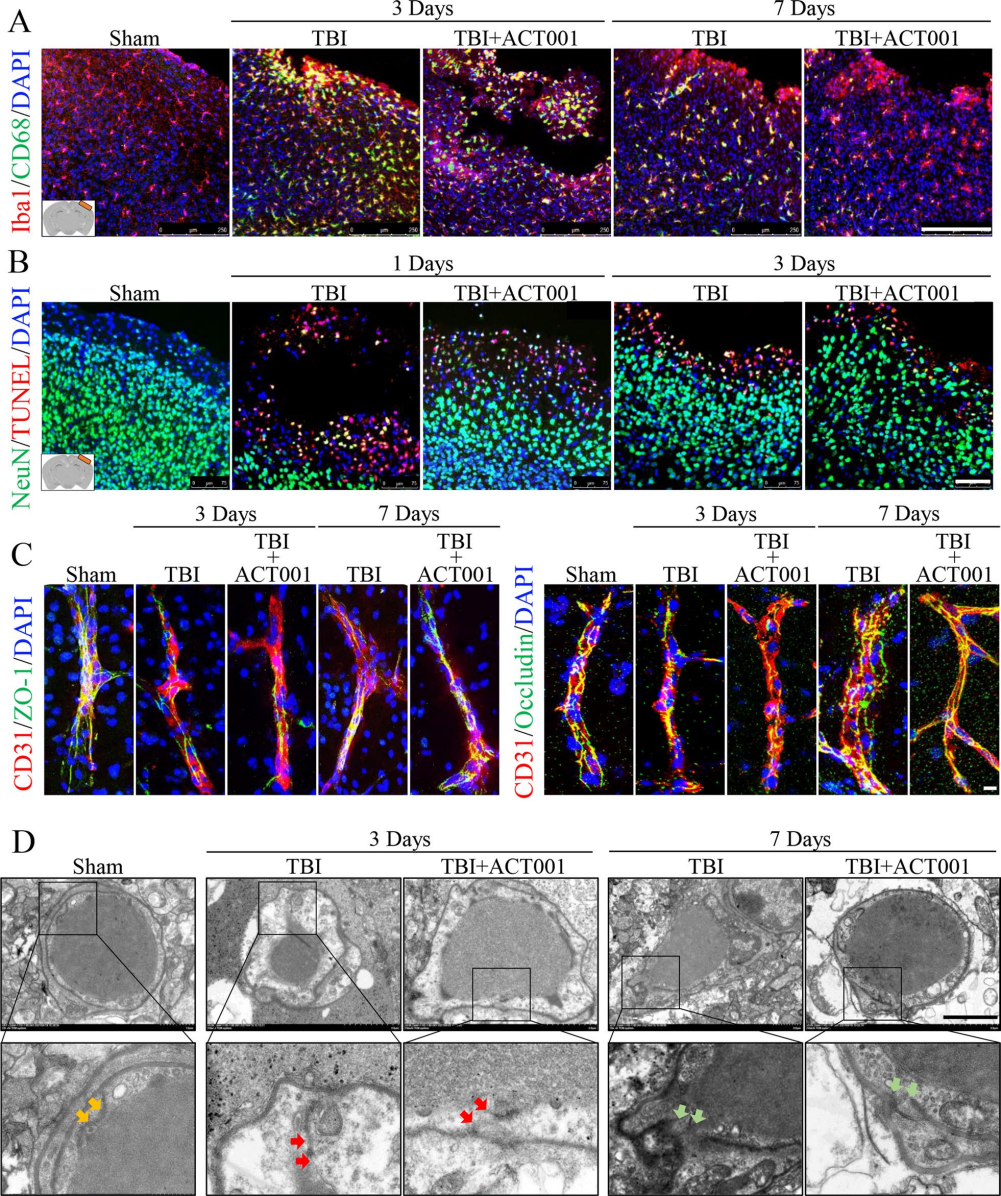
ACT001 alleviated microglial activation, decreased neuronal apoptosis and protected the continuity of tight junction proteins in BBB in vivo. **a** Representative fluorescence images for dual staining of Iba1 and CD68 in peri-contusion region from Sham, TBI and TBI + ACT001 groups at 3 and 7 days post-insult. Scale bar = 250 μm. **b** Representative fluorescence images of NeuN staining with TUNEL labelling in peri-contusion region from Sham, TBI and TBI + ACT001 groups at 1 and 3 days post-insult. Scale bar = 75 μm. **c** Representative fluorescence images for dual staining of CD31 and ZO-1, as well as CD31 and Occludin, in peri-contusion region from Sham, TBI and TBI + ACT001 groups at 3 and 7 days post-insult. Scale bar = 200 μm. Cell nuclei were shown in blue (DAPI) in (**a**–**c**). **d** Representative transmission electron microscope images of BBB structure in peri-contusion region from Sham, TBI and TBI + ACT001 groups at 3 and 7 days post-insult. The ultrastructure of BBB in Sham group was normal, the tight junction (orange arrows) of endothelium was compacted and continuous. In mice CCI models, contusion injury disrupted BBB integrity seriously, characterized by translucent cytoplasm, swelling mitochondria and membrane breakdown of endothelium. The tight junction was debonding bead-like (red arrows) at 3 days post-insult in TBI and TBI + ACT001 groups. After ACT001 treatment for 7 days, the BBB integrity (green arrows) in TBI + ACT001 group improved obviously compared with TBI group. Scale bar = 2 μm

Furthermore, in order to investigate precise role of ACT001 treatment in TBI, CD68 immunoreactivity in microglia cells (Iba1^+^) was used to evaluate trauma-induced microglial activation (Fig. 2a) [54]. TUNEL positive staining in neurons (NeuN^+^) was used to estimate cell apoptosis (Fig. 2b). Besides, tight junction proteins (ZO-1 and Occludin) expression in cerebral microvessels (CD31^+^) and TEM analysis were used to detect functional integrity of BBB (Fig. 2c, d). The results revealed that the number of Iba1^+^/CD68^+^ cells (*P* < 0.01, Fig. 2a and Additional file 4: Fig. S1C) and NeuN^+^/TUNEL^+^ cells (*P* < 0.001, Fig. 2b and Additional file 4: Fig. S1D) reduced gradually near contusional area in ACT001 + TBI group over time. ACT001 partly attenuated ZO-1 and Occludin mediated gaps formation (*P* < 0.01, Fig. 2c and Additional file 4: Fig. S1E) and improved the continuity of tight junction proteins in BBB (Fig. 2d) at 7 days post-TBI.

Taken together, these findings suggested that ACT001 treatment had therapeutic effects on CCI models and was beneficial for recovery after TBI in vivo.

### Delayed depletion of microglia cells with PLX5622 attenuated the efficacy of ACT001 in mice after TBI

**Fig. 3:**
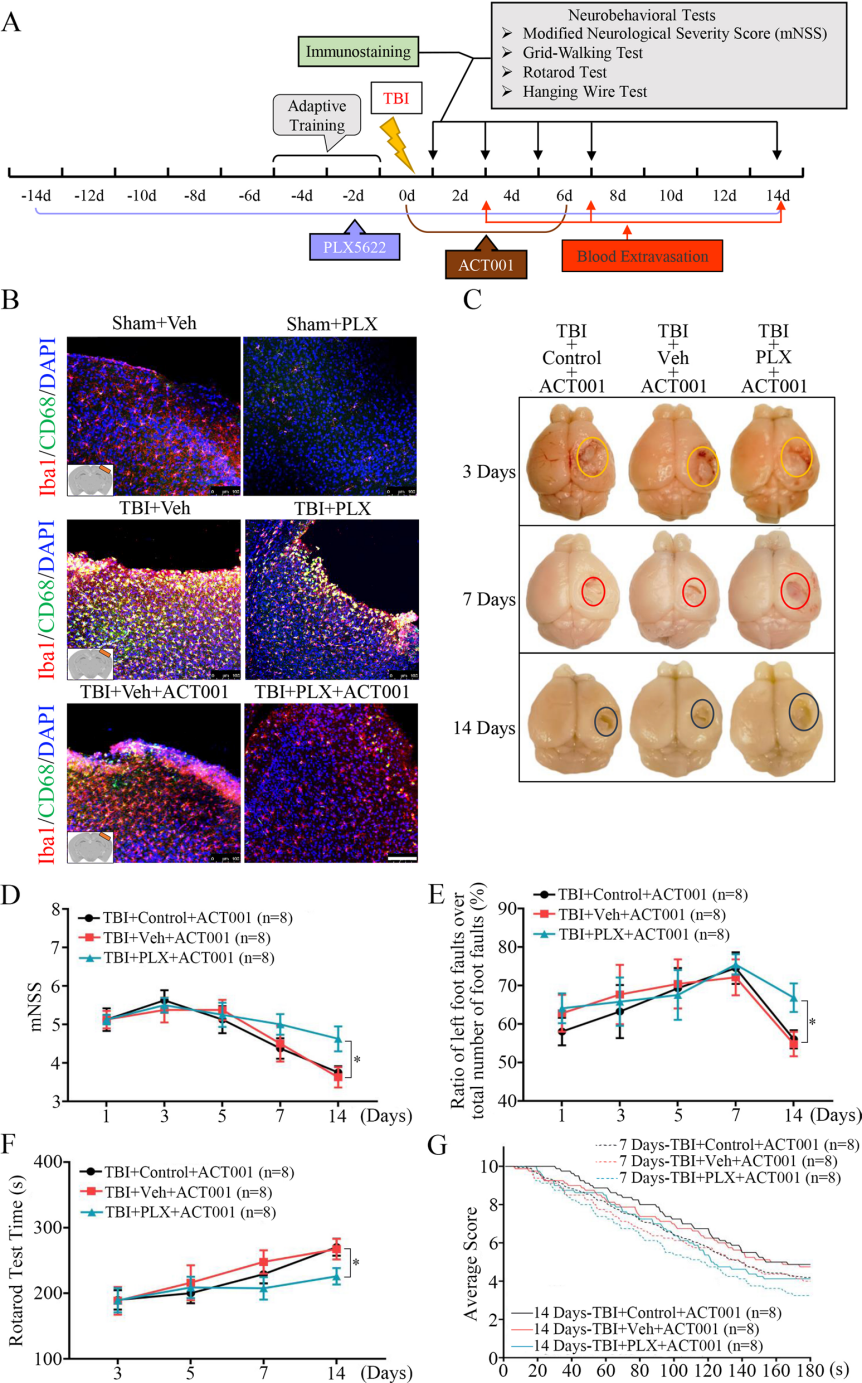
Elimination of microglia cells with the CSF1R antagonist PLX5622 attenuated therapeutic effects of ACT001 in mice CCI models. **a** Overview of experimental timeline for in vivo studies and neurobehavioral tests. Before CCI injury, the mice were provided diets formulated with either vehicle (Veh, AIN-76A chow) or PLX5622 (PLX) for 14 days. **b** Representative fluorescence images for dual staining of Iba1 and CD68 in peri-contusion region from Sham + Veh and Sham + PLX groups (up panel), TBI + Veh and TBI + PLX groups (middle panel), as well as TBI + Veh + ACT001 and TBI + PLX + ACT001 groups (bottom panel). Cell nuclei were shown in blue (DAPI). Scale bar = 100 μm. **c** Representative photographs of whole brains in TBI + Control + ACT001, TBI + Veh + ACT001 and TBI + PLX + ACT001 groups at 3 and 7 days post-insult. The circles represented the approximate area of lesion cortex. **d**–**g** Line graphs showed four neurobehavioral function assessments at indicated time points including mNNS scores (**d**), Grid-Walking test (**e**), Rotarod test (**f**) and Hanging Wire test (**g**). n = 8/group. Data were presented as mean ± SEMs, *P < 0.05, versus TBI + Veh + ACT001 group. TBI + Control + ACT001 group was the CCI models fed with normal chow and ACT001. TBI + Veh + ACT001 group was the CCI models fed with AIN-76A and ACT001. TBI + PLX + ACT001 group was the CCI models fed with PLX5622 and ACT001

Then we identified whether microglia cells absence altered the therapeutic effects of ACT001 after TBI in vivo. For this, we depleted microglia cells by feeding the mice with PLX5622 [55], which was able to achieve robust brain-wide microglial elimination with low side-effects on other CNS-resident cells [56, 57]. The experimental timeline was shown in Fig. 3a. Dietary administration of PLX5622 (Sham + PLX group), initiated 2 weeks prior to TBI, resulted in almost 95% depletion efficiency of microglia cells (Iba1^+^), which stood in sharp contrast to mice administered with AIN-76A chow (Sham + Veh group, Fig. 3b).

Having confirmed efficient depletion of brain microglia cells with PLX5622 administration, we next subjected the mice to CCI injury and ACT001 treatment. The results showed that TBI significantly promoted proliferation and activation of microglia cells in TBI + Veh group and PLX5622 could partly reverse this situation, leaving a few of the activated microglia cells to exist at the edge of lesion area (TBI + PLX group, Fig. 3b). Even though ACT001 inhibited TBI-induced microglial activation, delayed depletion of microglia cells still represented notable influence on drug efficacy in TBI + PLX + ACT001 group (*P* < 0.01, Additional file 4: Fig. S1F). The amount of extravasated blood presented in TBI + Control + ACT001 or TBI + Veh + ACT001 group was significantly less than that in TBI + PLX + ACT001 group at 7 and 14 days post-TBI, and there was no significant difference at 3 days (Fig. 3c).

As to neurobehavioral function recovery evaluation in mice, we first confirmed that microglial depletion did not affect animal behavior in non-injured mice (Additional file 4: Fig. S1G–S1J). After TBI, ACT001 treatment could still exert its neuroprotective function in TBI + Veh + ACT001 group. Similar results were also obtained from the CCI models fed with normal rodent chow (TBI + Control + ACT001 group). However, PLX5622 administration partly attenuated the efficacy of ACT001 treatment and exacerbated neurobehavioral deficiency in mNSS scores (*P* < 0.05, Fig. 3d), Grid-Walking test (*P* < 0.05, Fig. 3e), Rotarod test (*P* < 0.05, Fig. 3f) and Hanging Wire test (*P* < 0.001, Fig. 3g).

Collectively, these data indicated that the therapeutic effects of ACT001 in TBI might be related with the activation of microglia cells.

### ACT001 suppressed LPS-induced pro-inflammatory activation of BV2 cells

**Fig. 4:**
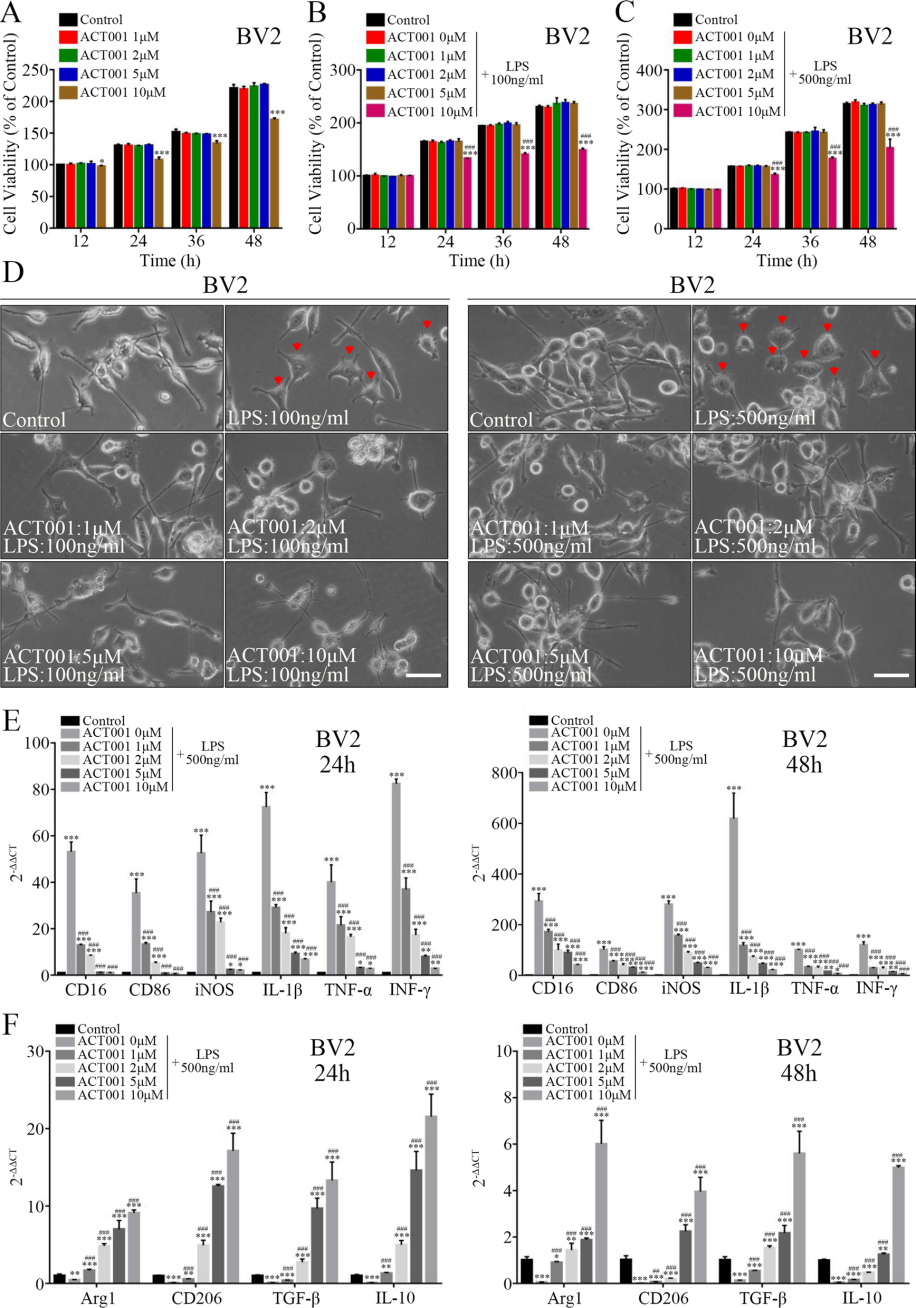
ACT001 mitigated LPS-induced pro-inflammatory activation of BV2 cells in vitro. **a** BV2 cells were treated with indicated doses of ACT001 for 12–48 h, then the cytotoxicity of ACT001 was measured by CCK-8 assay. The cell viability result was normalized to BV2 cells without ACT001 treatment (Control) for 12 h. **b, c** Indicated doses of ACT001 were co-treated with 100 ng/ml LPS (**b**) or 500 ng/ml LPS (**c**) in BV2 cells for 12–48 h, then the cytotoxicity of co-treatment was measured by CCK-8 assay. The cell viability result was normalized to BV2 cells without ACT001 and LPS treatment (Control) for 12 h. **d** The effect of ACT001 and LPS co-treatment on morphological changes of BV2 cells. The normal morphology of BV2 cells cultured in complete DMEM for 24 h was shown as Control. Red arrows indicated inflammatory amoeboid morphology of activated BV2 cells. Scale bar = 50 μm. **e, f** After co-treatment with indicated doses of ACT001 and 500 ng/ml LPS for 24 and 48 h, relative mRNA expression levels of pro-inflammatory cytokines (**e**) and anti-inflammatory cytokines (**f**) in BV2 cells were quantified by Real-time PCR. Cells without ACT001 and LPS treatment were shown as Control. Data were presented as means ± SEMs of three independent experiments. *P < 0.05, **P < 0.01, ***P < 0.001 versus Control group; # *P* < 0.05, ## *P* < 0.01, ### *P* < 0.001 versus ACT001 0 μM + LPS group

The aforementioned findings prompted us that ACT001 might possess inhibitory effect on microglia-mediated neuroinflammatory response in vivo. Therefore, we used LPS-induced microglial activation in vitro model to simulate trauma-induced condition in vivo as reported previously [58–60], which could further help to investigated whether its therapeutic effect was also a general function working in vitro. Prior to verify precise role of ACT001 in microglia cells, CCK-8 assay was performed to identify its cytotoxicity to BV2 cells. After treatment with different concentrations of ACT001 (ranging from 1 to 500 μM) for 12–48 h, we determined the safe concentration range of ACT001 was within 10 μM (Fig. 4a), and the cell viability of BV2 cells could significantly decrease in both dose- and time-dependent manner when ACT001 concentration exceeded 10 μM (Additional file 5: Fig. S2A). Thus, the concentrations of ACT001 ranging from 1 to 10 μM were used in subsequent experiments. Next, we investigated the inhibitory effects of ACT001 on LPS-induced microglial activation. The results of CCK-8 assay showed that the viability of BV2 cells increased moderately after being treated with 100 or 500 ng/ml LPS (Additional file 5: Fig. S2B), and this LPS-stimulated viability improvement was not altered significantly with gradient concentrations (within 10 μM) of ACT001 co-treatment for 12–48 h (Fig. 4b, c).

The morphological changes of BV2 cells were also evaluated after treatment with LPS and ACT001. As shown in Fig. 4d, classic BV2 cells grew with adherence, showing spindle shaped bodies with ramified morphology, characterized by small soma and distal arborization. LPS treatment (100 or 500 ng/ml) transformed BV2 cells into inflammatory amoeboid morphology, characterized by thick and short cell bodies. However, ACT001 could attenuate LPS-induced morphological changes of BV2 cells. Then, Real-time PCR was used to investigate whether ACT001 regulated the expression of inflammatory cytokines produced by BV2 cells. The results showed that the expression of pro-inflammatory cytokines increased significantly after 100 ng/ml (Additional file 5: Fig. S2C) or 500 ng/ml (Fig. 4e) LPS treatment for 24 h and 48 h, as evidenced by production of CD16, CD86, iNOS, IL-1β, TNF-α and INF-γ. While the level of anti-inflammatory cytokines, such as Arg1, CD206, TGF-β and IL-10, decreased significantly (Additional file 5: Fig. S2D and Fig. 4f). Interestingly, ACT001 treatment could suppress LPS-induced pro-inflammatory cytokines production and partly elevate anti-inflammatory cytokines synthesis in a dose-dependent manner (1–10 μM).

All results above indicated that ACT001 possessed potent anti-inflammatory effects on LPS-induced BV2 microglial activation.

### ACT001 moderated LPS-induced primary microglia activation

**Fig. 5:**
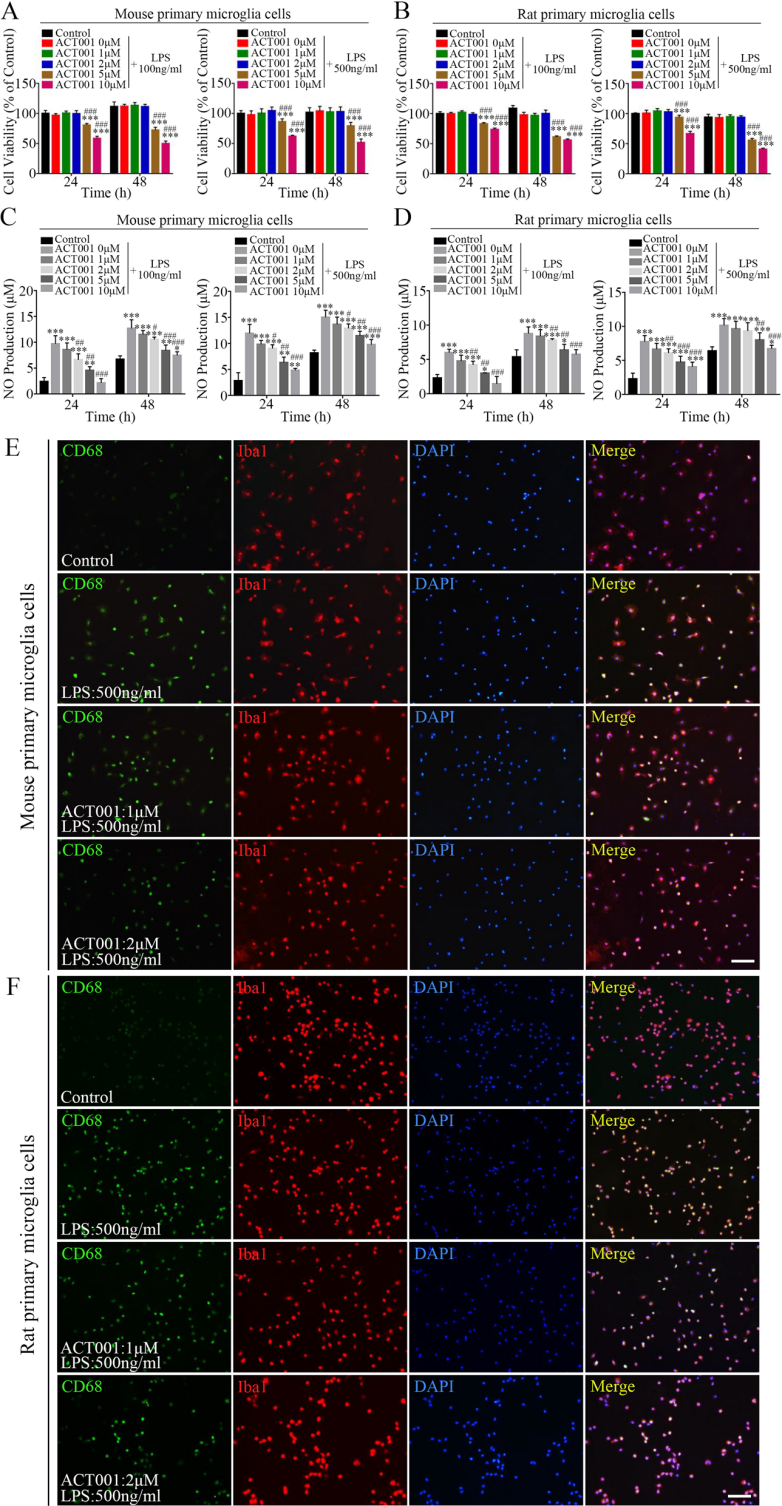
ACT001 exerted its inhibitory effect on LPS-induced primary microglia activation in vitro. **a, b** Indicated doses of ACT001 were co-treated with LPS in mouse (**a**) and rat (**b**) primary microglia cells for 24 and 48 h, then the cytotoxicity of co-treatment was measured by CCK-8 assay. The cell viability result was normalized to primary microglia cells without ACT001 and LPS treatment (Control) for 24 h. **c, d** After co-treatment with indicated doses of ACT001 and LPS for 24 and 48 h, the NO production levels in culture supernatants of mouse (**c**) and rat (**d**) primary microglia cells were determined using Griess reagent. **e, f** Representative fluorescence images for dual staining of Iba1 and CD68 in mouse (**e**) and rat (**f**) primary microglia cells after co-treatment with indicated doses of ACT001 and 500 ng/ml LPS for 24 h. Cell nuclei were shown in blue (DAPI). Scale bar = 400 μm. Cells without ACT001 and LPS treatment were shown as Control. Data were presented as means ± SEMs of three independent experiments. *P < 0.05, **P < 0.01, ***P < 0.001 versus Control group; # *P* < 0.05, ## *P* < 0.01, ### *P* < 0.001 versus ACT001 0 μM + LPS group

Primary microglia cells, which exhibit more complex biological characteristic than BV2 cells, are prevalent in neuroinflammatory research due to more similar phenotype to in vivo state [61]. Therefore, we next intended to verify whether ACT001 also exerted its therapeutic potential in primary microglia cells. We first subjected mouse and rat primary microglia cells to CCK-8 assay. The results showed that LPS treatment (100 or 500 ng/ml) had slight inhibitory effect on mouse and rat primary microglia cells (Additional file 6: Fig. S3A and S3B) and the cell viability was not altered significantly with (within 5 μM) ACT001 co-treatment for 12–48 h (Fig. 5a, b). Then, we examined whether ACT001 treatment could suppress LPS-stimulated NO production in primary microglia cells by Griess assay. As shown in Fig. 5c, d, compared with control group, NO production in mouse and rat primary microglia cells increased obviously after LPS treatment. Conversely, ACT001 co-incubation could decrease NO levels in a dose-dependent manner.

Next, the amounts of CD68 presented in Iba1^+^ microglia cells were used to assess microglial activation. The results showed that CD68^+^ cells were rare in control group, but were identified frequently after various concentrations of LPS treatment in mouse (Additional file 6: Fig. S3C and Fig. 5e) and rat primary microglia cells (Additional file 6: Fig. S3D and Fig. 5f). In contrast, accompanying with increased concentrations of ACT001 treatment, the proportion of CD68^+^ cells decreased gradually.

Based on these findings, LPS-induced mouse and rat primary microglia cells activation could also be alleviated by ACT001 treatment.

### ACT001 reduced neuronal apoptosis induced by activated microglia cells in vitro

**Fig. 6:**
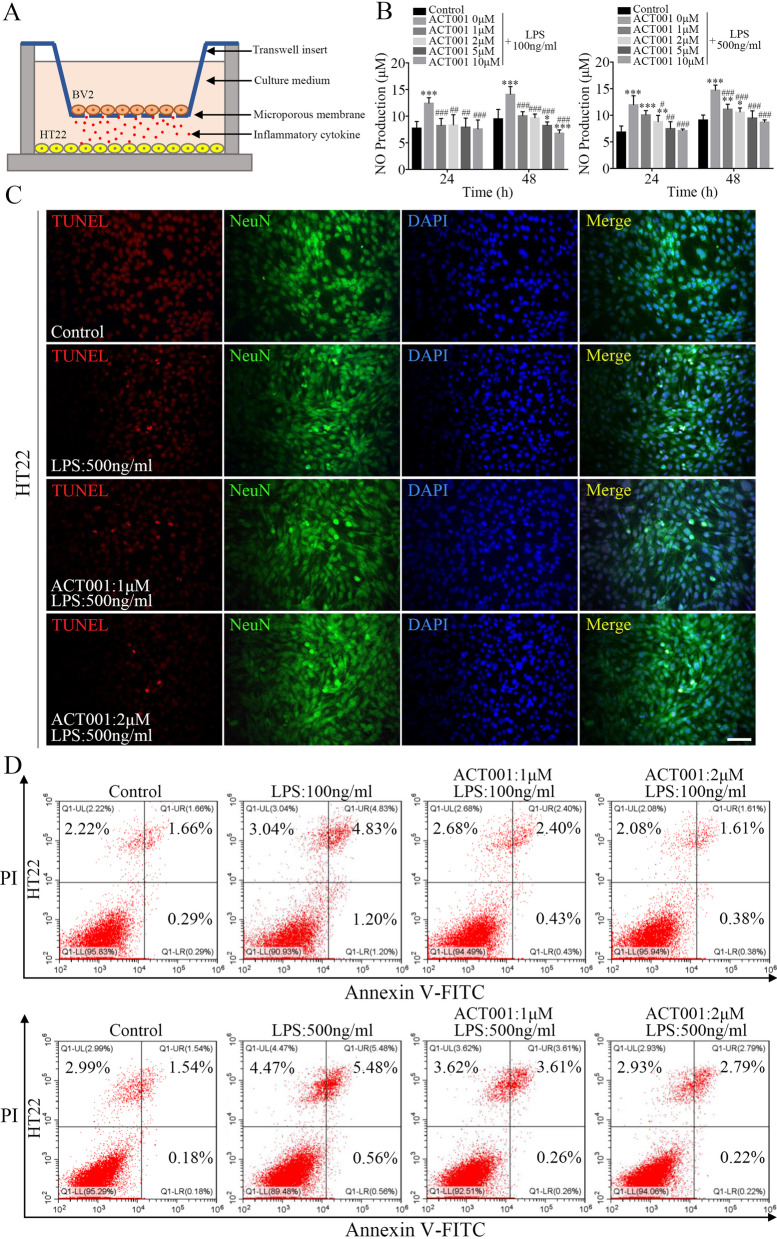
ACT001 reversed HT22 cells apoptosis induced by activated BV2 cells. **a** Schematic diagram of BV2-HT22 cell interaction model structure. BV2 cells were pre-treated with LPS for 24 h, then switched into models and co-cultured with HT22 cells in ACT001-added complete DMEM for 24 and 48 h. **b** After co-culturing for 24 and 48 h, the NO production levels in supernatants of cell interaction models were determined by using Griess reagent. **c** Representative fluorescence images of NeuN staining with TUNEL labelling in HT22 cells after co-culturing in models for 24 h. Cell nuclei were shown in blue (DAPI). Scale bar = 100 μm. **d** After co-culturing for 24 h, HT22 cells apoptosis were analyzed through flow cytometry assay by Annexin V-FITC/PI dual staining. Cell interaction models without ACT001 and LPS treatment were shown as Control. Data were presented as means ± SEMs of three independent experiments. *P < 0.05, **P < 0.01, ***P < 0.001 versus Control group; # *P* < 0.05, ## *P* < 0.01, ### *P* < 0.001 versus ACT001 0 μM + LPS group

Microglial activation state results in high-released levels of pro-inflammatory and cytotoxic mediators, which can induce delayed BBB repairment and neuronal apoptosis [10, 20, 21]. Next, we subjected LPS-stimulated BV2 and HT22 cells to cell-cell interaction models to further evaluate the effect of ACT001 on microglia-mediated neuronal apoptosis. First, we treated HT22 cells with LPS and ACT001 to identify drug cytotoxicity to cells viability. The results showed that the safe concentration range of ACT001 was within 10 μM (Additional file 7: Fig. S4A), and LPS treatment had mild inhibitory effect on HT22 cells (Additional file 7: Fig. S4B). Then, the cell-cell interaction models were set up to further study the effect of ACT001 (1–2 μM) on neuronal apoptosis due to the pro-inflammatory cytokines secreted by microglia cells. BV2 cells were cultured in upper chambers and pre-treated with LPS or PBS alone as control for 24 h, while HT22 cells were added into the bottom of 24-well plates. After that, upper chambers were switched to co-culture with HT22 cells in ACT001-added medium (without LPS) for 24–48 h (Fig. 6a). The culture supernatants were collected to determine NO levels. As shown in Fig. 6b, NO production in HT22 and LPS-stimulated BV2 cells co-cultured medium increased significantly as compared with control group. However, ACT001 could decrease LPS-induced NO levels in a dose-dependent manner. The apoptotic cells of HT22 were detected by TUNEL assay. Compared with control group, TUNEL-positive expression of HT22 cells (NeuN^+^) increased significantly after co-culturing with LPS-stimulated BV2 cells for 24 h (Additional file 7: Fig. S4C and Fig. 6c), and ACT001 could partly suppress cell apoptosis induced by activated BV2 cells. Similar results were also obtained from Annexin V-FITC/PI apoptosis assay, the numbers of both early and late apoptotic HT22 cells increased in LPS-stimulated BV2 cells co-cultured group, whereas ACT001 reversed the effect in a dose-dependent manner (Fig. 6d).

To conclude, these results suggested that HT22 cells apoptosis induced by activated BV2 microglia cells could be reversed by ACT001 treatment in vitro.

### ACT001 alleviated angiolysis mediated by activated microglia cells in vitro

**Fig. 7:**
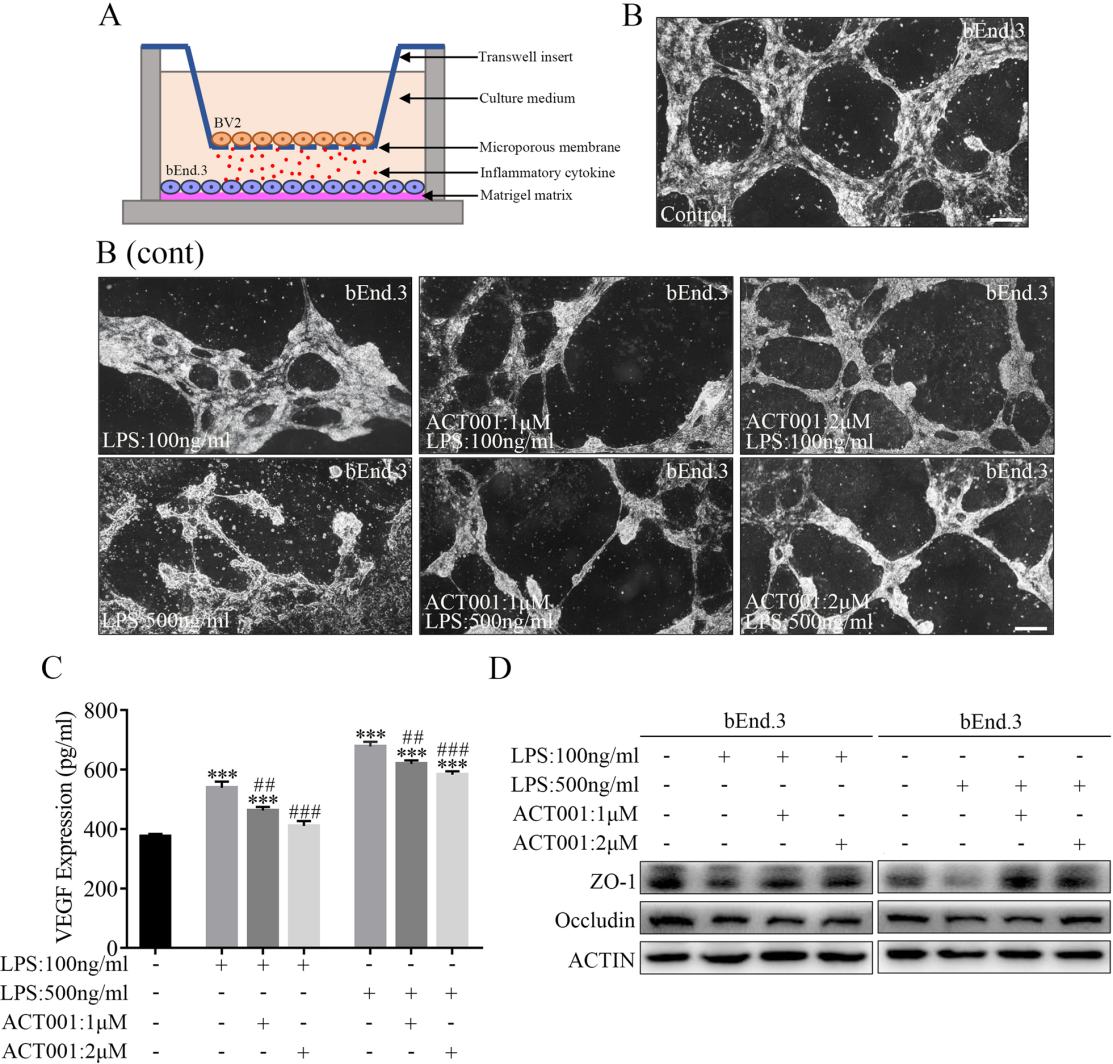
ACT001 promoted angiogenesis of bEnd.3 in cell interaction models. **a** Schematic diagram of BV2-bEnd.3 cell interaction model structure. bEnd.3 cells were seeded in each Matrigel-coated well and co-cultured with pre-stimulated BV2 cells for 24 h. **b** The tube formation of bEnd.3 cells after co-culturing with BV2 cells for 24 h without ACT001 and LPS treatment were shown as Control. Scale bar = 150 μm. **b** (cont) After co-culturing with 100 ng/ml LPS (B, up panel) or 500 ng/ml LPS (**b**, down panel) pre-stimulated BV2 cells for 24 h, the tube formation of bEnd.3 cells decreased markedly. Conversely, ACT001 treatment could reverse this effect and promote the angiogenesis in a dose-dependent manner. Scale bar = 150 μm. **c** The VEGF expression in bEnd.3 and  LPS-stimulated BV2 cells co-cultured medium was analyzed by ELISA assays. **d** Representative western blot results of ZO-1 and Occludin in bEnd.3 cells after co-culturing for 24 h. Actin was used as control for protein loading. * *P* < 0.05, **P < 0.01, ***P < 0.001 versus Con trol group; # *P* < 0.05, ## *P* < 0.01, ### *P* < 0.00 1 versus LPS group

Next, we investigated whether ACT001 treatment affected angiogenesis and angiolysis after microglia-mediated inflammatory response. First, we performed CCK-8 assay to verify the cytotoxicity of LPS and ACT001 treatment to bEnd.3 cells. The results showed that the safe concentration range of ACT001 was within 5 μM (Additional file 7: Fig. S4D), and LPS had inhibitory effect on bEnd.3 cells (Additional file 7: Fig. S4E). Then, bEnd.3 cells and LPS-stimulated BV2 cells were subjected to cell-cell interaction models and co-cultured in ACT001-added (1–2 μM) medium (without LPS) for 24 h (Fig. 7a). In tube formation assay, LPS-stimulated BV2 cells significantly inhibited tube formation capacity of bEnd.3 cells at 24 h as compared with control group. On the contrary, ACT001 treatment alleviated this angiolysis effect and partly promoted angiogenesis of bEnd.3 cells (Fig. 7b). In addition, ELISA assays showed that the concentration of VEGF increased significantly in bEnd.3 and LPS-stimulated BV2 cells co-cultured medium. Acting as a cytokine involved in inflammatory response, VEGF could induce tight junction disassembly and breakdown of endothelial permeability barrier by altering the organization of tight junction proteins, such as ZO-1 and Occludin [62–64]. However, ACT001 treatment could partly reverse this effect and decrease VEGF levels (Fig. 7c). Subsequently, western blot analyses also proved that ZO-1 and Occludin expression in bEnd.3 cells decreased initially after being co-cultured with LPS-stimulated BV2 cells for 24 h, and ACT001 treatment could restore the protein expression to a certain extent (Fig. 7d).

All these data indicated that activated BV2 microglia cells inhibited angiogenesis and reduced the tight junction proteins expression in bEnd.3 cells, and ACT001 could alleviate these effects in vitro.

### ACT001 suppressed NFκB/NLRP3 neuroinflammatory pathway by binding to AKT

**Fig. 8:**
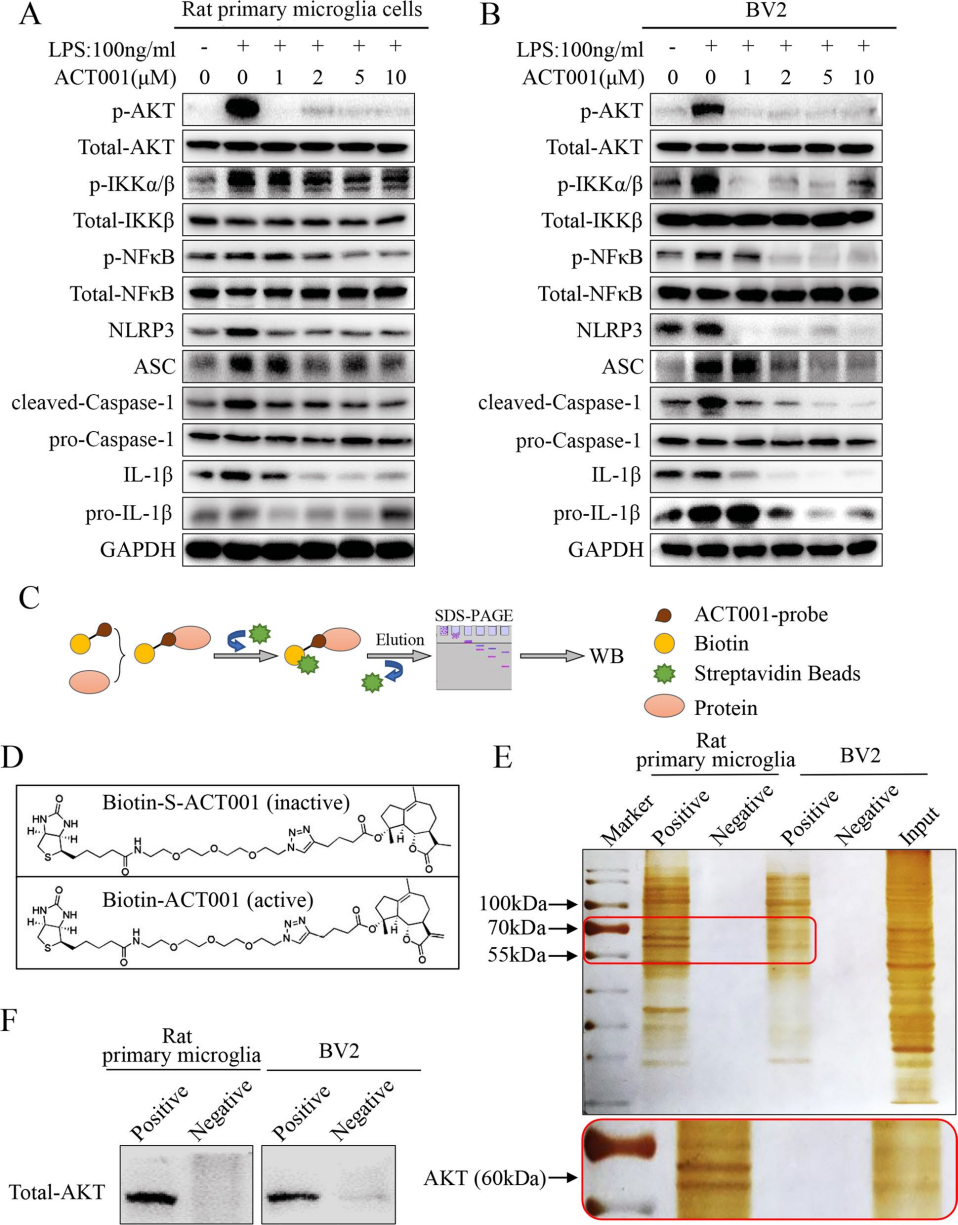
ACT001 suppressed NFκB/NLRP3 neuroinflammatory pathway by inhibiting the phosphorylation of AKT. **(a-b)** After co-treatment with indicated doses of ACT001 and 100 ng/ml LPS for 24 h, rat primary microglia cells (**a**) and BV2 cells (**b**) were subjected to immunoblot analysis for p-AKT/Total-AKT, p-IKKα/β/Total-IKKβ, p-NFκB/Total-NFκB, NLRP3, ASC, cleaved-Caspase-1/pro-Caspase-1, IL-1β/pro-IL-1β expression, and GAPDH was used as c ontrol for protein loading. **c** Schematic diagram of ACT001 probe pull-down experiment. **d** Chemical structure of ACT001-S-biotin probe (inactive) and ACT001-biotin probe (active) were shown as indicated. **e** Proteins pulled down by the probe in rat primary microglia cells and BV2 cells were detected through silver staining. Input referred to the whole protein lysates from these two cells; negative referred to ACT001-S-biotin probe solution; and positive referred to proteins pulled down by ACT001-biotin probe. **f** Proteins precipitated by ACT001-biotin probe or ACT001-S-biotin probe in rat primary microglia cells and BV2 cells were detected by western blotting using an anti-Total-AKT primary antibody

NFκB/NLRP3 pathway played an important role in regulating microglia-mediated neuroinflammatory response after TBI [65–67]. In order to further clarify the underlying mechanism of ACT001 treatment, we subjected rat primary microglia cells and BV2 cells to western blotting assays. In accordance with previous studies, we found that the expression levels of p-IKKα/β, p-NFκB, NLRP3, ASC, cleaved-Caspase-1 and IL-1β notably increased after LPS treatment. However, accompanying with increasing concentrations of ACT001 treatment, the proteins expression decreased gradually in rat primary microglia cells (Fig. 8a) and BV2 cells (Fig. 8b). Besides, ACT001 could markedly reverse the nuclear translocation of NFκB induced by LPS treatment in mouse and rat primary microglia cells (Additional file 8: Fig. S5A and S5B). Specifically, the phosphorylation level of AKT, acting as an upstream regulatory protein to NFκB [68], showed remarkable changes after LPS and ACT001 treatment. Therefore, we isolated ACT001-biotin-bound proteins as described previously to verify the interaction between ACT001 and NFκB/NLRP3 pathway [38, 52] (Fig. 8c). The active probe and inactive probe of ACT001 were designed and synth esized (Fig. 8d). The proteins pulled down by probe in rat primary microglia cells and BV2 cells were further visualized by silver staining. The results showed positive bands at approximately 70–55 kDa, which corresponded to molecular weight of AKT (60 kDa, Fig. 8e). Furthermore, western blotting assays also confirmed that AKT was one of proteins precipitated by biotin-ACT001 but not biotin-S-ACT001, illustrating that AKT might interact with ACT001 in rat primary microglia cells and BV2 cells.

Based on these findings, we determined that ACT001 inhibited the nuclear translocation of NFκB and further suppressed NLRP3 pathway activation via decreasing the phosphorylation of AKT.

## Discussion

Previous research has indicated that ACT001 possessed anti-inflammatory properties in multiple diseases and cancer treatment [29, 53, 69–71] with little toxicity and few side effects. However, whether ACT001 also exerts its therapeutic effects on microglia-mediated neuroinflammatory response following TBI is unclear. In this study, we demonstrated for the first time that ACT001 treatment relieved neurological impairment and facilitated recovery process in mice CCI models, indicating that ACT001 had potential clinical application for TBI. In addition, microglial elimination by PLX5622 could restrain the pharmacological action of ACT001 dramatically in vivo, which further verified that microglia cells might be the target cells to ACT001 treatment, and the drug efficacy was associated with increased microglial activity.

TBI was peculiarly challenging to treat because of its heterogeneous nature and complex pathological mechanism. Recently, neuroinflammation has been recognized as an important molecular and cellular characteristic following TBI [10, 65], further exacerbating mechanical insult (primary injury) and initiating neuronal cell death and BBB disruption [8, 9]. Microglia, acting as primary mediators of innate immune response in CNS, play critical roles in neuroinflammation and secondary injury [11, 15, 16]. Even though activated microglia cells are beneficial at acute phase of TBI, these advantages could be countered when the activation is uninterrupted [10, 56]. CNS injuries trigger a series of injurious biochemical cascades and disinhibited reactive microglial activation state resulting in high-released levels of pro-inflammatory and cytotoxic mediators [10]. Our research suggested that ACT001 possessed potential anti-inflammatory effects on BV2 and primary microglia cells (derived from mice and rats) activation. Meanwhile, ACT001 could effectively inhibit LPS-induced pro-inflammatory cytokines release (CD16, CD86, iNOS, IL-1β, TNF-α, INF-γ) in BV2 cells, which were also considered as “M1-like” microglial phenotype markers, and partly promote the expression of some “M2-like” microglial phenotype markers, such as Arg1, CD206, TGF-β and IL-10. This was a very interesting phenomenon worthy of attention. We speculated that the alternation of “M1/M2-like” paradigm induced by ACT001 in microglia cells was the major influential factor explaining its therapeutic effect on trauma-induced neuronal apoptosis and angiolysis.

In studies involving microglial activation, “M1/M2-like” paradigm debate has always been an inevitable “hot-button” issue. These two distinct phenotypes were first theorized based on the original finding that IL-4 mediated inflammatory macrophages (M1-like) adopted an alternative activation phenotype with reduced pro-inflammatory cytokines secretion (M2-like) [72]. However, with the development of biomedical study, researchers later recognized that microglia cells had distinctive lineage and molecular signatures compared with bone-marrow derived macrophages [73, 74]. “M1/M2-like” phenotypes only represented two major extreme types of activated microglia cells, and this simple division standard has been challenged [75]. In mice CCI model, microglia cells could concurrently express both M1- and M2-like phenotype markers on the same cell at multiple time points [76]. Besides, there was no clear transformation form between M1- and M2-like microglia cells [77, 78]. In this regard, we proposed that any therapeutic strategy that targeted trauma-induced microglial activation should be fine-tuned from “M1/M2-like” paradigm debate to selectively suppress “danger signaling pathways” (i.e., pro-inflammatory response) and/or promote “healthy signaling pathways” (i.e., anti-inflammatory response) [65, 79].

**Fig. 9:**
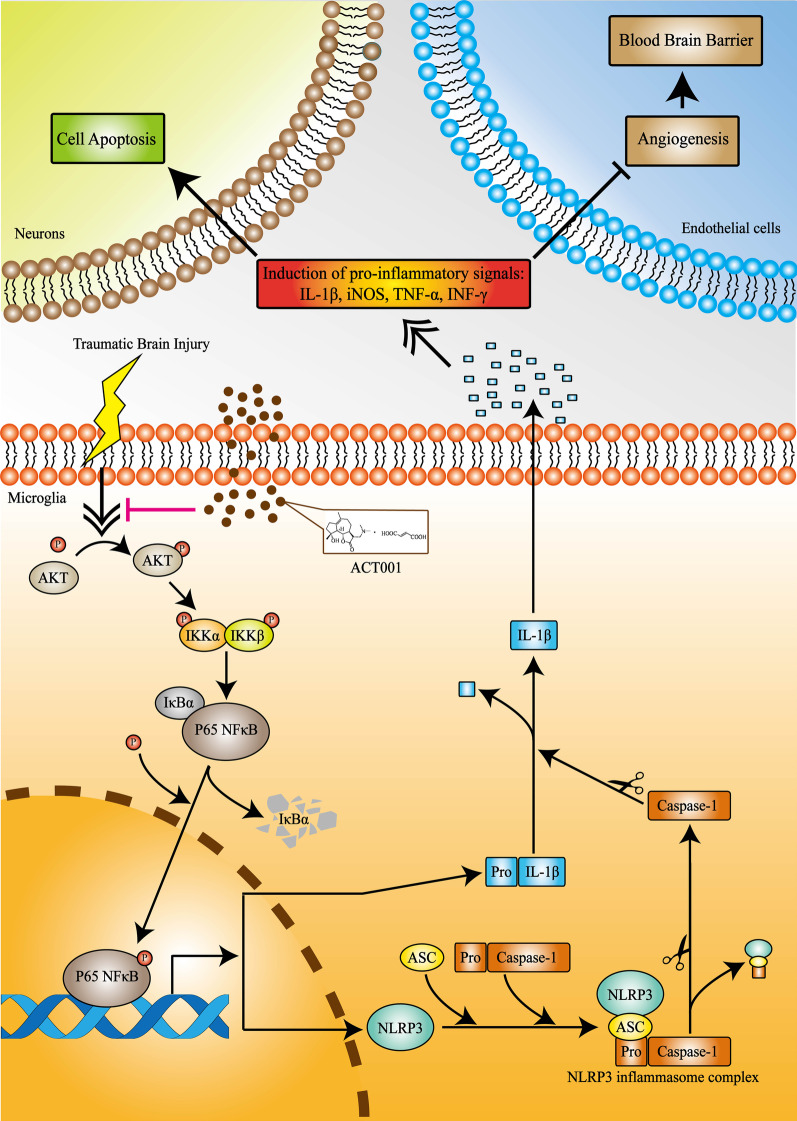
Schematic illustration depicting that ACT001 attenuated NLRP3 neuroinflammatory pathway in microglia cells through restraining NFκB nuclear translocation by inhibiting the phosphorylation of AKT

Among these “danger signaling pathways”, NFκB/NLRP3 inflammasome, which has been documented to regulate inflammatory activation of glial cells (including astrocytes and microglia cells), is considered as a main causative factor of neuroinflammation and neurological dysfunction following TBI [66]. NLRP3 inflammasome mainly consists of three protein subunits: NLRP3, which is a cytosolic sensor molecule, an adaptor protein containing a caspase activating recruitment domain named ASC, and an effector protein acting as a cysteine protease, pro-Caspase-1 [80]. The activation of NLRP3 inflammasome required a two-checkpoint signal process. The initial external stimulation, such as TBI, promoted NFκB activation and translocation into the nucleus and further enhanced transcriptional expression of inflammasome components. This process was called priming signal. Next, subsequent (or prolonged) stimulation induced assembly of these components into complete NLRP3 inflammasome, and it further cleaved pro-Caspase-1 into its active isomer. Caspase-1 then facilitated the maturation and release of IL-1β, which played an important role in innate immune response to trauma and creating generalized pro-inflammatory environment. This process was called activation signal [80]. In this study, we found that ACT001 treatment could inhibit nuclear translocation of NFκB in microglia cells and arrest NLRP3 inflammasome formation in priming signal stage. Subsequent pull-down and western blotting assays further indicated that ACT001 decreased the phosphorylation level of AKT, an upstream kinase catalyzing the phosphorylation of IKKα/β and IκBα, resulting in the deactivation of NFκB [81, 82] (Fig. 9).

## Conclusions

In summary, combined with data from previous studies regarding the properties of ACT001 in CNS diseases [28, 36, 38, 39] such as the characteristics of minimal adverse effects and high BBB permeability, our study uncovered that ACT001 could attenuate microglia-mediated neuroinflammation and improve recovery of neurological deficits following TBI, and the associated mechanism was related to its inhibitory effect on AKT phosphorylation, which could further restrain NFκB nuclear translocation and suppress NLRP3 inflammasome formation. These findings provided us with novel insight into the mechanism of ACT001 function, as well as its potential therapeutic effect on TBI. Follow-up clinical trials would be necessary to further evaluate its treatment value in TBI.

## Supplementary Information

**Additional file 1: Table S1**. Reagents used in this study.

**Additional file 2: Table S2**. The detailed criteria of neurobehavioral function assessments.

**Additional file 3: Table S3**. Nucleotide Sequences of the Real-time PCR Primers.

**Additional file 4: Fig. S1** (A) Statistical results of cresyl violet-stained brain sections at indicated time points post-insult in mice CCI models. n = 6/group. (B) Statistical results of EB extravasation in brain tissue at indicated time points post-insult in mice CCI models. n = 6/group. (C) Statistical results of CD68+ cells in microglia cells (Iba1+) at indicated time points post-insult in mice CCI models. (D) Statistical results of apoptotic cells (Tunel+) in neurons (NeuN+) at indicated time points post-insult in mice CCI models. (E) Statistical results of ZO-1+ (left) or Occludin+ (right) cells in cerebral microvessels (CD31+) at indicated time points post-insult in mice CCI models. (F) Statistical results of CD68+ cells in microglia cells (Iba1+) at indicated time points post-insult in mice CCI models (fed with PLX5622 and ACT001). (G-J) Bar graphs showed four neurobehavioral function assessments at indicated time points including mNNS scores (G), Grid-Walking test (H), Rotarod test (I) and Hanging Wire test (J). n = 8/group. The results showed that no statistical difference was found between Vehicle group (non-injured mice provided diets formulated with AIN-76A chow) and PLX5622 group (non-injured mice provided diets formulated with PLX5622). Data were presented as mean ± SEMs. **P < 0.01, ***P < 0.001  versus TBI group or TBI + Veh + ACT001 group.

**Additional file 5: Fig. S2** (A) BV2 cells were treated with indicated doses of ACT001 for 12-48 hours, then the cytotoxicity of ACT001 was measured by CCK-8 assay. The cell viability result was normalized to BV2 cells without ACT001 treatment ( Control) for 12 hours. (B) BV2 cells were treated with indicated doses of LPS for 12-48 hours, then the cytotoxicity of LPS was measured by CCK-8 assay. The cell viability result was normalized to BV2 cells with 100 ng/ml LPS treatment for 12 hours. (C-D) After co-treatment with indicated doses of ACT001 and 100 ng/ml LPS for 24 and 48 hours, relative mRNA expression levels of pro-inflammatory cytokines (C) and anti-inflammatory cytokines (D) i n BV2 cells were quantified by Real-time PCR. Cells without ACT001 and LPS treatment were shown as control. Data were prese nted as means ± SEMs of three independent experiments. *P < 0.05, **P < 0.01, ***P < 0.001 versus Control group or 100 ng/ml LPS group; # *P* < 0 . 0 5, ## *P* < 0.01 , ### *P* < 0.001 versus ACT001 0 μM + LPS group.

**Additional file 6: Fig. S3** (A-B) Mouse (A) and rat (B) primary microglia cells were treated with indicated doses of LPS for 24 and 48 hours, then the cytotoxicity of LPS was measured by CCK-8 assay. The cell viability result was normalized to cells with 100 ng/ml LPS treatment for 24 hours. (C-D) Representative fluorescence images for dual staining of Iba-1 and CD68 in mouse (C) and rat (D) primary microglia cells after co-treatment with indicated doses of ACT001 and 100 ng/ml LPS for 24 hours. Cell nuclei were shown in blue (DAPI). Scale bar = 400 μm. Cells without ACT001 and LPS treatment were shown as Control. Data were presented as means ± SEMs of three independent experiments. *P < 0.05, **P < 0.01, ***P < 0.001 versus 100 ng/ml LPS group.

**Additional file 7: Fig. S4** (A) HT22 cells were treated with indicated doses of ACT001 for 12-48 hours, then the cytotoxicity of ACT001 was measured by CCK-8 assay. The cell viability result was normalized to HT22 cells without ACT001 treatment (Control) for 12 hours. (B) HT22 cells were treated with indicated doses of LPS for 12-48 hours, then the cytotoxicity of LPS was measured by CCK-8 assay. The cell viability result was normalized to HT22 cells with 100 ng/ml LPS treatment for 12 hours. (C) Representative fluorescence images of NeuN staining with TUNEL labelling in HT22 cells after co-culturing in models for 24 hours. Cell nuclei were shown in blue (DAPI). Scale bar = 100 μm. (D) bEnd.3 cells were treated with indicated doses of ACT001 for 12-48 hours, then the cytotoxicity of ACT001 was measured by CCK-8 assay. The cell viability result was normalized to bEnd.3 cells without ACT001 treatment (Control) for 12 hours. (E) bEnd.3 cells were treated with indicated doses of LPS for 12-48 hours, then the cytotoxicity of LPS was measured by CCK-8 assay. The cell viability result was normalized to bEnd.3 cells with 100 ng/ml LPS treatment for 12 hours. Data were presented as means ± SEMs of three independent experiments. **P < 0.01, ***P < 0.001 versus Control group or 100 ng/ml LPS group.

**Additional file 8: Fig. S5** (A-B) Representative fluorescence images for dual staining of NFkB and Iba1 in mouse (A) and rat (B) primary microglia cells after co-treatment with indicated doses of ACT001 and 100 ng/ml LPS for 24 hours. Cell nuclei were shown in blue (DAPI). Scale bar = 10 μm. Cells without ACT001 and LPS treatment were shown as control.

## Data Availability

The datasets used and/or analyzed during the current study are available from the corresponding author on reasonable request.

## Abbreviations

TBI: Traumatic brain injury
CNS: Central nervous system
BBB: Blood–brain barrier
CCI: Controlled cortical impact
CSF1R: Colony stimulating factor 1 receptor
LPS: Lipopolysaccharides
MCL: Micheliolide
DMAMCL: Dimethylamino-micheliolide
FDA: Food and Drug Administration
SLs: Sesquiterpene lactones
GBM: Glioblastoma multiforme
CCTCC: China Center for Type Culture Collection
ATCC: American Type Culture Collection
DMEM: Dulbecco’s modified Eagle medium
FBS: Fetal bovine serum
PLL: Poly-ℒ-lysine-coated
PBS: Phosphate buffered saline
PFA: Paraformaldehyde
EB: Evans blue
mNSS: Modified Neurological Severity Score
Rotor-Rod: Rotarod Test
BSA: Bovine serum albumin
TUNEL: Terminal dexynucleotidyl transferase mediated dUTP nick end labeling
TEM: Transmission electron microscopy
CCK-8: Cell Counting kit-8
NO: Nitric oxide
ANOVA: One-way analysis of variance

## References

[1] Menon DK, Schwab K, Wright DW, Maas AI, Health DaCAWGotIaIItCDEfRoTBIaP. Position statement: definition of traumatic brain injury. Arch Phys Med Rehabil. 2010;91:1637–1640. 10.1016/j.apmr.2010.05.017 21044706

[2] Mollayeva T, Mollayeva S, Colantonio A (2018). Traumatic brain injury: sex, gender and intersecting vulnerabilities. Nat Rev Neurol.

[3] Yang DX, Jing Y, Liu YL, Xu ZM, Yuan F, Wang ML (2019). Inhibition of transient receptor potential vanilloid 1 attenuates blood-brain barrier disruption after traumatic brain injury in mice. J Neurotrauma.

[4] Smith DH, Meaney DF, Shull WH (2003). Diffuse axonal injury in head trauma. J Head Trauma Rehabil.

[5] Humble SS, Wilson LD, Wang L, Long DA, Smith MA, Siktberg JC (2018). Prognosis of diffuse axonal injury with traumatic brain injury. J Trauma Acute Care Surg.

[6] Stoica BA, Faden AI (2010). Cell death mechanisms and modulation in traumatic brain injury. Neurotherapeutics.

[7] Xu X, Gao W, Cheng S, Yin D, Li F, Wu Y (2017). Anti-inflammatory and immunomodulatory mechanisms of atorvastatin in a murine model of traumatic brain injury. J Neuroinflammation.

[8] Capizzi A, Woo J, Verduzco-Gutierrez M (2020). Traumatic brain injury: an overview of epidemiology, pathophysiology, and medical management. Med Clin North Am.

[9] Shively SB, Priemer DS, Stein MB, Perl DP (2021). Pathophysiology of traumatic brain injury, chronic traumatic encephalopathy, and neuropsychiatric clinical expression. Psychiatr Clin North Am.

[10] Loane DJ, Kumar A (2016). Microglia in the TBI brain: the good, the bad, and the dysregulated. Exp Neurol.

[11] Donat CK, Scott G, Gentleman SM, Sastre M (2017). Microglial activation in traumatic brain injury. Front Aging Neurosci.

[12] Simon DW, McGeachy MJ, Bayir H, Clark RSB, Loane DJ, Kochanek PM (2017). The far-reaching scope of neuroinflammation after traumatic brain injury. Nat Rev Neurol.

[13] Roberts I, Yates D, Sandercock P, Farrell B, Wasserberg J, Lomas G (2004). Effect of intravenous corticosteroids on death within 14 days in 10008 adults with clinically significant head injury (MRC CRASH trial): randomised placebo-controlled trial. Lancet.

[14] Skolnick BE, Maas AI, Narayan RK, van der Hoop RG, MacAllister T, Ward JD (2014). A clinical trial of progesterone for severe traumatic brain injury. N Engl J Med.

[15] Hanisch UK, Kettenmann H (2007). Microglia: active sensor and versatile effector cells in the normal and pathologic brain. Nat Neurosci.

[16] Panday A, Inda ME, Bagam P, Sahoo MK, Osorio D, Batra S (2016). Transcription factor NF-kappaB: an update on intervention strategies. Arch Immunol Ther Exp (Warsz).

[17] Nathalie M, Polineni SP, Chin CN, Fawcett D, Clervius H, Maria QSL (2021). Targeting microglial polarization to improve TBI outcomes. CNS Neurol Disord Drug Targets.

[18] Brown GC, Vilalta A, Fricker M (2015). Phagoptosis—cell death by phagocytosis—plays central roles in physiology, host defense and pathology. Curr Mol Med.

[19] Ramlackhansingh AF, Brooks DJ, Greenwood RJ, Bose SK, Turkheimer FE, Kinnunen KM (2011). Inflammation after trauma: microglial activation and traumatic brain injury. Ann Neurol.

[20] Shlosberg D, Benifla M, Kaufer D, Friedman A (2010). Blood-brain barrier breakdown as a therapeutic target in traumatic brain injury. Nat Rev Neurol.

[21] Cherry JD, Tripodis Y, Alvarez VE, Huber B, Kiernan PT, Daneshvar DH (2016). Microglial neuroinflammation contributes to tau accumulation in chronic traumatic encephalopathy. Acta Neuropathol Commun.

[22] Gasparini C, Feldmann M (2012). NF-kappaB as a target for modulating inflammatory responses. Curr Pharm Des.

[23] Mincheva-Tasheva S, Soler RM (2013). NF-kappaB signaling pathways: role in nervous system physiology and pathology. Neuroscientist.

[24] Dresselhaus EC, Meffert MK (2019). Cellular specificity of NF-kappaB function in the nervous system. Front Immunol.

[25] Zhang Q, Lu Y, Ding Y, Zhai J, Ji Q, Ma W (2012). Guaianolide sesquiterpene lactones, a source to discover agents that selectively inhibit acute myelogenous leukemia stem and progenitor cells. J Med Chem.

[26] An Y, Guo W, Li L, Xu C, Yang D, Wang S (2015). Micheliolide derivative DMAMCL inhibits glioma cell growth in vitro and in vivo. PLoS ONE.

[27] Jaffar J, Glaspole I, Symons K, Westall G (2021). Inhibition of NF-kappaB by ACT001 reduces fibroblast activity in idiopathic pulmonary fibrosis. Biomed Pharmacother.

[28] Liu Q, Zhang S, Zhu D, Tang X, Che Y, Feng X (2020). The parthenolide derivative ACT001 synergizes with low doses of L-DOPA to improve MPTP-induced Parkinson's disease in mice. Behav Brain Res.

[29] Viennois E, Xiao B, Ayyadurai S, Wang L, Wang PG, Zhang Q (2014). Micheliolide, a new sesquiterpene lactone that inhibits intestinal inflammation and colitis-associated cancer. Lab Investig.

[30] Ghantous A, Gali-Muhtasib H, Vuorela H, Saliba NA, Darwiche N (2010). What made sesquiterpene lactones reach cancer clinical trials?. Drug Discov Today.

[31] Ivanescu B, Miron A, Corciova A (2015). Sesquiterpene lactones from artemisia genus: biological activities and methods of analysis. J Anal Methods Chem.

[32] Hehner SP, Heinrich M, Bork PM, Vogt M, Ratter F, Lehmann V (1998). Sesquiterpene lactones specifically inhibit activation of NF-kappa B by preventing the degradation of I kappa B-alpha and I kappa B-beta. J Biol Chem.

[33] Saadane A, Masters S, DiDonato J, Li J, Berger M (2007). Parthenolide inhibits IkappaB kinase, NF-kappaB activation, and inflammatory response in cystic fibrosis cells and mice. Am J Respir Cell Mol Biol.

[34] Jin P, Madieh S, Augsburger LL (2007). The solution and solid state stability and excipient compatibility of parthenolide in feverfew. AAPS PharmSciTech.

[35] Xi XN, Liu N, Wang QQ, Wu HT, He HB, Wang LL (2019). Pharmacokinetics, tissue distribution and excretion of ACT001 in Sprague-Dawley rats and metabolism of ACT001. J Chromatogr B Analyt Technol Biomed Life Sci.

[36] Li Q, Sun Y, Liu B, Li J, Hao X, Ge W (2020). ACT001 modulates the NF-kappaB/MnSOD/ROS axis by targeting IKKbeta to inhibit glioblastoma cell growth. J Mol Med (Berl).

[37] Lickliter JD, Jennens R, Lemech CR, Su SYC, Chen Y (2018). Phase 1 dose-escalation study of ACT001 in patients with recurrent glioblastoma and other advanced solid tumors. J Clin Oncol.

[38] Xi X, Liu N, Wang Q, Chu Y, Yin Z, Ding Y, Lu Y (2019). ACT001, a novel PAI-1 inhibitor, exerts synergistic effects in combination with cisplatin by inhibiting PI3K/AKT pathway in glioma. Cell Death Dis.

[39] Tong L, Li J, Li Q, Wang X, Medikonda R, Zhao T (2020). ACT001 reduces the expression of PD-L1 by inhibiting the phosphorylation of STAT3 in glioblastoma. Theranostics.

[40] Tamashiro TT, Dalgard CL, Byrnes KR. Primary microglia isolation from mixed glial cell cultures of neonatal rat brain tissue. J Vis Exp. 2012:e3814. 10.3791/3814 PMC3486750 22929966

[41] Du S, Xiong S, Du X, Yuan TF, Peng B, Rao Y. Primary Microglia Isolation from Postnatal Mouse Brains. J Vis Exp. 2021. 10.3791/62237 33720125

[42] Schwulst SJ, Islam M. Murine model of controlled cortical impact for the induction of traumatic brain injury. J Vis Exp. 2019. 10.3791/60027 PMC7046175 31475969

[43] Horvath RJ, Nutile-McMenemy N, Alkaitis MS, Deleo JA (2008). Differential migration, LPS-induced cytokine, chemokine, and NO expression in immortalized BV-2 and HAPI cell lines and primary microglial cultures. J Neurochem.

[44] Das A, Kim SH, Arifuzzaman S, Yoon T, Chai JC, Lee YS (2016). Transcriptome sequencing reveals that LPS-triggered transcriptional responses in established microglia BV2 cell lines are poorly representative of primary microglia. J Neuroinflammation.

[45] Zhang Z, Zhang L, Zhou C, Wu H (2014). Ketamine inhibits LPS-induced HGMB1 release in vitro and in vivo. Int Immunopharmacol.

[46] Rios EC, de Lima TM, Moretti AI, Soriano FG (2016). The role of nitric oxide in the epigenetic regulation of THP-1 induced by lipopolysaccharide. Life Sci.

[47] Gu C, Wang F, Zhang YT, Wei SZ, Liu JY, Sun HY (2021). Microglial MT1 activation inhibits LPS-induced neuroinflammation via regulation of metabolic reprogramming. Aging Cell.

[48] Han X, Xu T, Fang Q, Zhang H, Yue L, Hu G, Sun L (2021). Quercetin hinders microglial activation to alleviate neurotoxicity via the interplay between NLRP3 inflammasome and mitophagy. Redox Biol.

[49] Morizawa YM, Hirayama Y, Ohno N, Shibata S, Shigetomi E, Sui Y (2017). Reactive astrocytes function as phagocytes after brain ischemia via ABCA1-mediated pathway. Nat Commun.

[50] Shi X, Luo L, Wang J, Shen H, Li Y, Mamtilahun M (2021). Stroke subtype-dependent synapse elimination by reactive gliosis in mice. Nat Commun.

[51] Wu ZB, Cai L, Lin SJ, Leng ZG, Guo YH, Yang WL (2016). Heat shock protein 47 promotes glioma angiogenesis. Brain Pathol.

[52] Li J, Li S, Guo J, Li Q, Long J, Ma C (2018). Natural product micheliolide (MCL) irreversibly activates pyruvate kinase m2 and suppresses leukemia. J Med Chem.

[53] Liu Q, Guo X, Huang Z, He Q, Zhu D, Zhang S, et al. Anti-neuroinflammatory effects of dimethylaminomylide (DMAMCL, i.e., ACT001) are associated with attenuating the NLRP3 in fl ammasome in MPTP-induced Parkinson disease in mice. Behav Brain Res. 2020;383:112539. 10.1016/j.bbr.2020.112539 32032741

[54] Hoogland IC, Houbolt C, van Westerloo DJ, van Gool WA, van de Beek D (2015). Systemic inflammation and microglial activation: systematic review of animal experiments. J Neuroinflammation.

[55] Elmore MR, Najafi AR, Koike MA, Dagher NN, Spangenberg EE, Rice RA (2014). Colony-stimulating factor 1 receptor signaling is necessary for microglia viability, unmasking a microglia progenitor cell in the adult brain. Neuron.

[56] Bellver-Landete V, Bretheau F, Mailhot B, Vallieres N, Lessard M, Janelle ME (2019). Microglia are an essential component of the neuroprotective scar that forms after spinal cord injury. Nat Commun.

[57] Willis EF, MacDonald KPA, Nguyen QH, Garrido AL, Gillespie ER, Harley SBR, et al. Repopulating Microglia Promote Brain Repair in an IL-6-Dependent Manner. Cell. 2020;180:833–846 e816. 10.1016/j.cell.2020.02.013 32142677

[58] Loane DJ, Stoica BA, Pajoohesh-Ganji A, Byrnes KR, Faden AI (2009). Activation of metabotropic glutamate receptor 5 modulates microglial reactivity and neurotoxicity by inhibiting NADPH oxidase. J Biol Chem.

[59] Loane DJ, Stoica BA, Byrnes KR, Jeong W, Faden AI (2013). Activation of mGluR5 and inhibition of NADPH oxidase improves functional recovery after traumatic brain injury. J Neurotrauma.

[60] Kumar A, Henry RJ, Stoica BA, Loane DJ, Abulwerdi G, Bhat SA, Faden AI (2019). Neutral sphingomyelinase inhibition alleviates lps-induced microglia activation and neuroinflammation after experimental traumatic brain injury. J Pharmacol Exp Ther.

[61] Stansley B, Post J, Hensley K (2012). A comparative review of cell culture systems for the study of microglial biology in Alzheimer's disease. J Neuroinflammation.

[62] Wang Wen, Dentler William L., Borchardt Ronald T. (2001). VEGF increases BMEC monolayer permeability by affecting occludin expression and tight junction assembly. American Journal of Physiology-Heart and Circulatory Physiology.

[63] Wang Li-Feng, Li Xiang, Gao Ya-Bing, Wang Shui-Ming, Zhao Li, Dong Ji, Yao Bin-Wei, Xu Xin-Ping, Chang Gong-Min, Zhou Hong-Mei, Hu Xiang-Jun, Peng Rui-Yun (2015). Activation of VEGF/Flk-1-ERK Pathway Induced Blood–Brain Barrier Injury After Microwave Exposure. Molecular Neurobiology.

[64] Inada M, Xu H, Takeuchi M, Ito M, Chen M. Microglia increase tight-junction permeability in coordination with Muller cells under hypoxic condition in an in vitro model of inner blood-retinal barrier. Exp Eye Res. 2021;205:108490. 10.1016/j.exer.2021.108490 33607076

[65] Jassam YN, Izzy S, Whalen M, McGavern DB, El Khoury J (2017). Neuroimmunology of traumatic brain injury: time for a paradigm shift. Neuron.

[66] O'Brien WT, Pham L, Symons GF, Monif M, Shultz SR, McDonald SJ (2020). The NLRP3 inflammasome in traumatic brain injury: potential as a biomarker and therapeutic target. J Neuroinflamm.

[67] Ismael S, Ahmed HA, Adris T, Parveen K, Thakor P, Ishrat T (2021). The NLRP3 inflammasome: a potential therapeutic target for traumatic brain injury. Neural Regen Res.

[68] Ozes ON, Mayo LD, Gustin JA, Pfeffer SR, Pfeffer LM, Donner DB (1999). NF-kappaB activation by tumour necrosis factor requires the Akt serine-threonine kinase. Nature.

[69] Sun Z, Li G, Tong T, Chen J (2017). Micheliolide suppresses LPS-induced neuroinflammatory responses. PLoS ONE.

[70] Zhang Q, Jiang X, He W, Wei K, Sun J, Qin X (2017). MCL plays an anti-inflammatory role in mycobacterium tuberculosis-induced immune response by inhibiting NF-kappaB and NLRP3 inflammasome activation. Mediators Inflamm.

[71] Liu W, Chen X, Wang Y, Chen Y, Chen S, Gong W (2019). Micheliolide ameliorates diabetic kidney disease by inhibiting Mtdh-mediated renal inflammation in type 2 diabetic db/db mice. Pharmacol Res.

[72] Stein M, Keshav S, Harris N, Gordon S (1992). Interleukin 4 potently enhances murine macrophage mannose receptor activity: a marker of alternative immunologic macrophage activation. J Exp Med.

[73] Ginhoux F, Greter M, Leboeuf M, Nandi S, See P, Gokhan S (2010). Fate mapping analysis reveals that adult microglia derive from primitive macrophages. Science.

[74] Gomez Perdiguero E, Klapproth K, Schulz C, Busch K, Azzoni E, Crozet L (2015). Tissue-resident macrophages originate from yolk-sac-derived erythro-myeloid progenitors. Nature.

[75] Ransohoff RM (2016). A polarizing question: do M1 and M2 microglia exist?. Nat Neurosci.

[76] Morganti JM, Riparip LK, Rosi S (2016). Call off the Dog(ma): M1/M2 polarization is concurrent following traumatic brain injury. PLoS ONE.

[77] Hu X, Li P, Guo Y, Wang H, Leak RK, Chen S (2012). Microglia/macrophage polarization dynamics reveal novel mechanism of injury expansion after focal cerebral ischemia. Stroke.

[78] Jin X, Ishii H, Bai Z, Itokazu T, Yamashita T (2012). Temporal changes in cell marker expression and cellular infiltration in a controlled cortical impact model in adult male C57BL/6 mice. PLoS ONE.

[79] Zhao SC, Ma LS, Chu ZH, Xu H, Wu WQ, Liu F (2017). Regulation of microglial activation in stroke. Acta Pharmacol Sin.

[80] Sutterwala FS, Haasken S, Cassel SL (2014). Mechanism of NLRP3 inflammasome activation. Ann N Y Acad Sci.

[81] Ji W, Liang K, An R, Wang X. Baicalin protects against ethanol-induced chronic gastritis in rats by inhibiting Akt/NF-kappaB pathway. Life Sci. 2019;239:117064. 10.1016/j.lfs.2019.117064 31734260

[82] Bang E, Kim DH, Chung HY. Protease-activated receptor 2 induces ROS-mediated inflammation through Akt-mediated NF-kappaB and FoxO6 modulation during skin photoaging. Redox Biol. 2021;44:102022. 10.1016/j.redox.2021.102022 PMC8182111 34082382

