# Supplementary material for: ACT001 attenuates microglia-mediated neuroinflammation after traumatic brain injury via inhibiting AKT/NFκB/NLRP3 pathway
Source: Cell Commun Signal. 2022 Apr 23;20:56. doi: 10.1186/s12964-022-00862-y (PMC9035258; doi:10.1186/s12964-022-00862-y)
Supplement: Supplementary file 5 — Additional file 4: Fig. S1 (A) Statistical results of cresyl violet-stained brain sections at indicated time points post-insult in mice CCI models. n = 6/group. (B) Statistical results of EB extravasation in brain tissue at indicated time points post-insult in mice CCI models. n = 6/group. (C) Statistical results of CD68+ cells in microglia cells (Iba1+) at indicated time points post-insult in mice CCI models. (D) Statistical results of apoptotic cells (Tunel+) in neurons (NeuN+) at indicated time points post-insult in mice CCI models. (E) Statistical results of ZO-1+ (left) or Occludin+ (right) cells in cerebral microvessels (CD31+) at indicated time points post-insult in mice CCI models. (F) Statistical results of CD68+ cells in microglia cells (Iba1+) at indicated time points post-insult in mice CCI models (fed with PLX5622 and ACT001). (G-J) Bar graphs showed four neurobehavioral function assessments at indicated time points including mNNS scores (G), Grid-Walking test (H), Rotarod test (I) and Hanging Wire test (J). n = 8/group. The results showed that no statistical difference was found between Vehicle group (non-injured mice provided diets formulated with AIN-76A chow) and PLX5622 group (non-injured mice provided diets formulated with PLX5622). Data were presented as mean ± SEMs. **P < 0.01, ***P < 0.001 versus TBI group or TBI + Veh + ACT001 group. [file 12964_2022_862_MOESM5_ESM.docx]

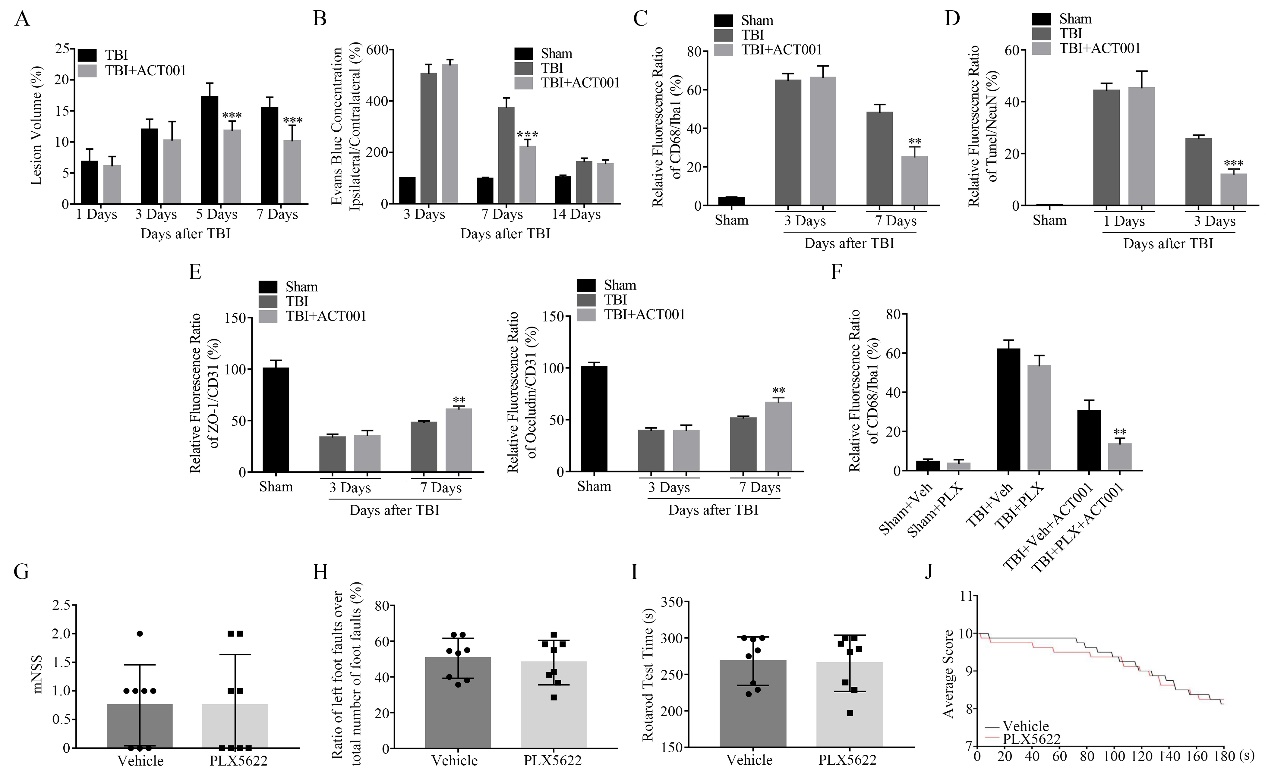


Supplemental Fig. 1 **(A)** Statistical results of cresyl violet-stained brain sections at indicated time points post-insult in mice CCI models. n = 6/group. **(B)** Statistical results of EB extravasation in brain tissue at indicated time points post-insult in mice CCI models. n = 6/group. **(C)** Statistical results of CD68^+^ cells in microglia cells (Iba1^+^) at indicated time points post-insult in mice CCI models. **(D)** Statistical results of apoptotic cells (Tunel^+^) in neurons (NeuN^+^) at indicated time points post-insult in mice CCI models. **(E)** Statistical results of ZO-1^+^ (left) or Occludin^+^ (right) cells in cerebral microvessels (CD31^+^) at indicated time points post-insult in mice CCI models. **(F)** Statistical results of CD68^+^ cells in microglia cells (Iba1^+^) at indicated time points post-insult in mice CCI models (fed with PLX5622 and ACT001). **(G-J)** Bar graphs showed four neurobehavioral function assessments at indicated time points including mNNS scores (G), Grid-Walking test (H), Rotarod test (I) and Hanging Wire test (J). n = 8/group. The results showed that no statistical difference was found between Vehicle group (non-injured mice provided diets formulated with AIN-76A chow) and PLX5622 group (non-injured mice provided diets formulated with PLX5622). Data were presented as mean ± SEMs. ****P* < 0.05, ***P* < 0.01, vs. TBI group or TBI+Veh+ACT001 group.
