# Supplementary material for: ACT001 attenuates microglia-mediated neuroinflammation after traumatic brain injury via inhibiting AKT/NFκB/NLRP3 pathway
Source: Cell Commun Signal. 2022 Apr 23;20:56. doi: 10.1186/s12964-022-00862-y (PMC9035258; doi:10.1186/s12964-022-00862-y)
Supplement: Supplementary file 6 — Additional file 5: Fig. S2 (A) BV2 cells were treated with indicated doses of ACT001 for 12-48 hours, then the cytotoxicity of ACT001 was measured by CCK-8 assay. The cell viability result was normalized to BV2 cells without ACT001 treatment ( Control) for 12 hours. (B) BV2 cells were treated with indicated doses of LPS for 12-48 hours, then the cytotoxicity of LPS was measured by CCK-8 assay. The cell viability result was normalized to BV2 cells with 100 ng/ml LPS treatment for 12 hours. (C-D) After co-treatment with indicated doses of ACT001 and 100 ng/ml LPS for 24 and 48 hours, relative mRNA expression levels of pro-inflammatory cytokines (C) and anti-inflammatory cytokines (D) i n BV2 cells were quantified by Real-time PCR. Cells without ACT001 and LPS treatment were shown as control. Data were prese nted as means ± SEMs of three independent experiments. *P < 0.05, **P < 0.01, ***P < 0.001 versus Control group or 100 ng/ml LPS group; # P < 0 . 0 5, ## P < 0.01 , ### P < 0.001 versus ACT001 0 μM + LPS group. [file 12964_2022_862_MOESM6_ESM.docx]

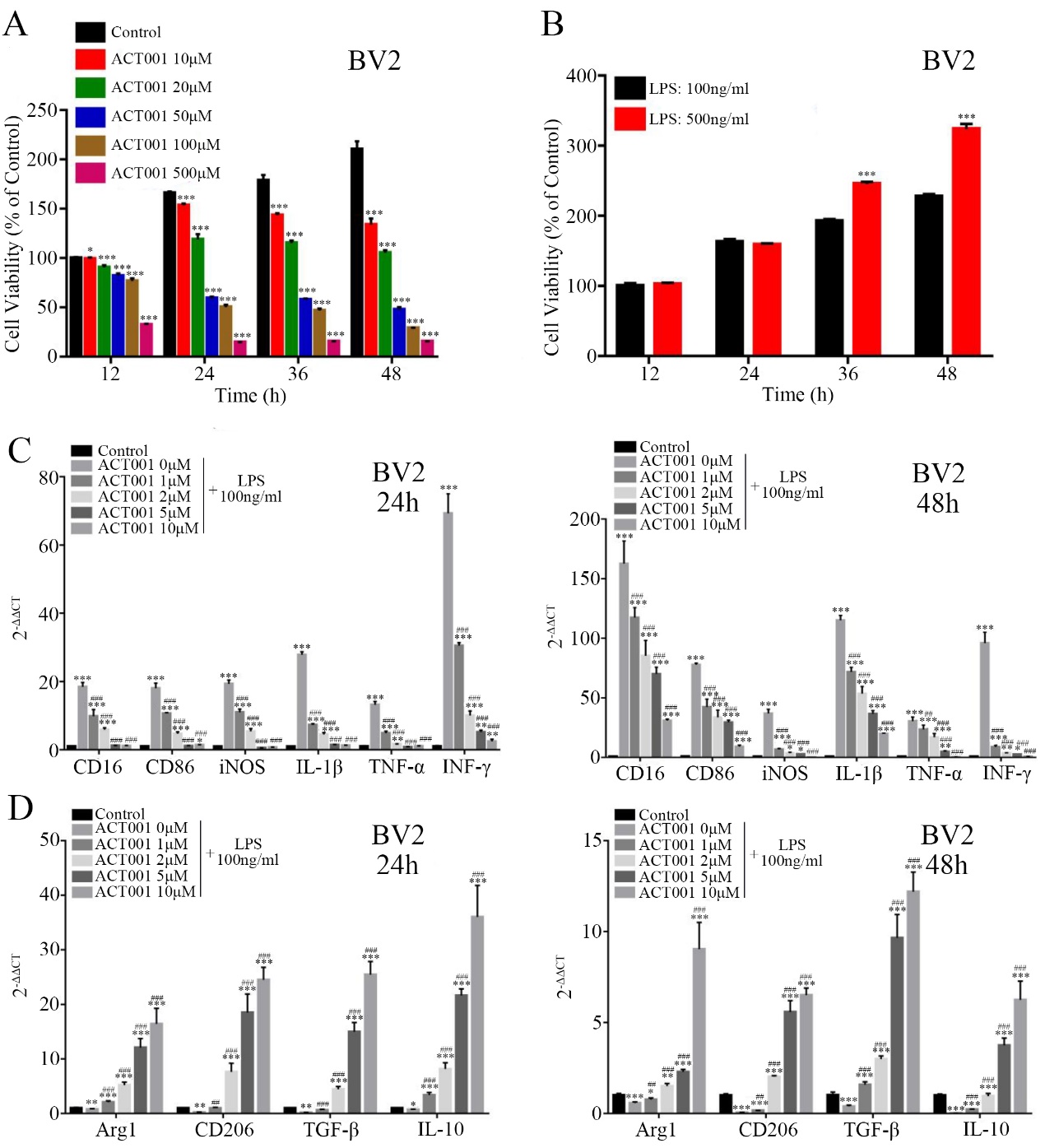


Supplemental Fig. 2 **(A)** BV2 cells were treated with indicated doses of ACT001 for 12-48 hours, then the cytotoxicity of ACT001 was measured by CCK-8 assay. The cell viability result was normalized to BV2 cells without ACT001 treatment (control) for 12 hours. **(B)** BV2 cells were treated with indicated doses of LPS for 12-48 hours, then the cytotoxicity of LPS was measured by CCK-8 assay. The cell viability result was normalized to BV2 cells with 100 ng/ml LPS treatment for 12 hours. **(C-D)** After co-treatment with indicated doses of ACT001 and 100 ng/ml LPS for 24 and 48 hours, relative mRNA expression levels of pro-inflammatory cytokines (C) and anti-inflammatory cytokines (D) in BV2 cells were quantified by Real-time PCR. Cells without ACT001 and LPS treatment were shown as control. Data were presented as means ± SEMs of three independent experiments. **P* < 0.05, ***P* < 0.01, ****P* < 0.001 vs. control group or 100 ng/ml LPS group; # *P* < 0.05, ## *P* < 0.01, ### *P* < 0.001 vs. ACT001 0 μM + LPS group.
