# Supplementary material for: ACT001 attenuates microglia-mediated neuroinflammation after traumatic brain injury via inhibiting AKT/NFκB/NLRP3 pathway
Source: Cell Commun Signal. 2022 Apr 23;20:56. doi: 10.1186/s12964-022-00862-y (PMC9035258; doi:10.1186/s12964-022-00862-y)
Supplement: Supplementary file 7 — Additional file 6: Fig. S3 (A-B) Mouse (A) and rat (B) primary microglia cells were treated with indicated doses of LPS for 24 and 48 hours, then the cytotoxicity of LPS was measured by CCK-8 assay. The cell viability result was normalized to cells with 100 ng/ml LPS treatment for 24 hours. (C-D) Representative fluorescence images for dual staining of Iba-1 and CD68 in mouse (C) and rat (D) primary microglia cells after co-treatment with indicated doses of ACT001 and 100 ng/ml LPS for 24 hours. Cell nuclei were shown in blue (DAPI). Scale bar = 400 μm. Cells without ACT001 and LPS treatment were shown as Control. Data were presented as means ± SEMs of three independent experiments. *P < 0.05, **P < 0.01, ***P < 0.001 versus 100 ng/ml LPS group. [file 12964_2022_862_MOESM7_ESM.docx]

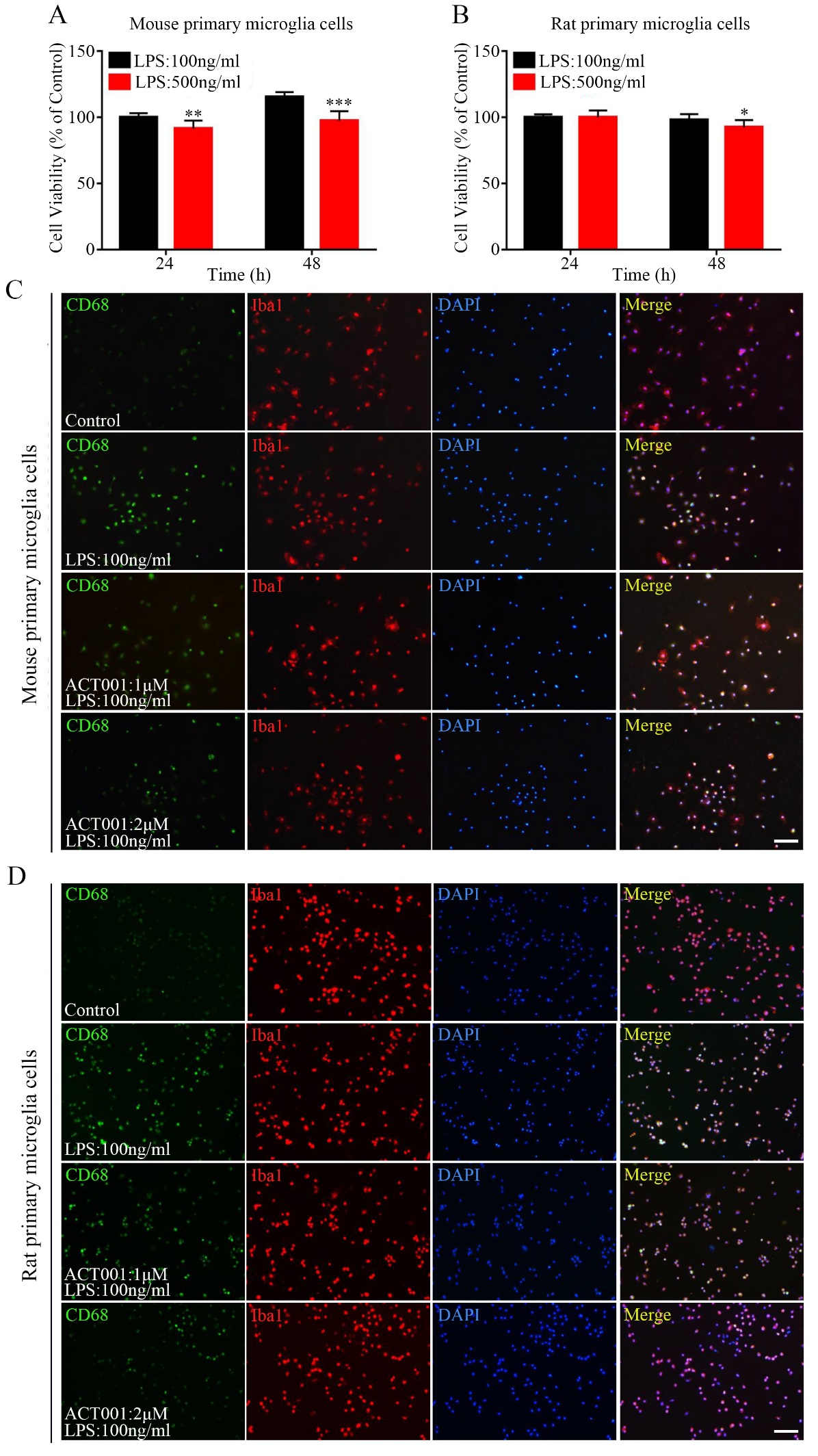


Supplemental Fig. 3 **(A-B)** Mouse (A) and rat (B) primary microglia cells were treated with indicated doses of LPS for 24 and 48 hours, then the cytotoxicity of LPS was measured by CCK-8 assay. The cell viability result was normalized to cells with 100 ng/ml LPS treatment for 24 hours. **(C-D)** Representative fluorescence images for dual staining of Iba-1 and CD68 in mouse (C) and rat (D) primary microglia cells after co-treatment with indicated doses of ACT001 and 100 ng/ml LPS for 24 hours. Cell nuclei were shown in blue (DAPI). Scale bar = 400 μm. Cells without ACT001 and LPS treatment were shown as control. Data were presented as means ± SEMs of three independent experiments. **P* < 0.05, ***P* < 0.01, ****P* < 0.001 vs. 100 ng/ml LPS group.
