# Supplementary material for: ACT001 attenuates microglia-mediated neuroinflammation after traumatic brain injury via inhibiting AKT/NFκB/NLRP3 pathway
Source: Cell Commun Signal. 2022 Apr 23;20:56. doi: 10.1186/s12964-022-00862-y (PMC9035258; doi:10.1186/s12964-022-00862-y)
Supplement: Supplementary file 8 — Additional file 7: Fig. S4 (A) HT22 cells were treated with indicated doses of ACT001 for 12-48 hours, then the cytotoxicity of ACT001 was measured by CCK-8 assay. The cell viability result was normalized to HT22 cells without ACT001 treatment (Control) for 12 hours. (B) HT22 cells were treated with indicated doses of LPS for 12-48 hours, then the cytotoxicity of LPS was measured by CCK-8 assay. The cell viability result was normalized to HT22 cells with 100 ng/ml LPS treatment for 12 hours. (C) Representative fluorescence images of NeuN staining with TUNEL labelling in HT22 cells after co-culturing in models for 24 hours. Cell nuclei were shown in blue (DAPI). Scale bar = 100 μm. (D) bEnd.3 cells were treated with indicated doses of ACT001 for 12-48 hours, then the cytotoxicity of ACT001 was measured by CCK-8 assay. The cell viability result was normalized to bEnd.3 cells without ACT001 treatment (Control) for 12 hours. (E) bEnd.3 cells were treated with indicated doses of LPS for 12-48 hours, then the cytotoxicity of LPS was measured by CCK-8 assay. The cell viability result was normalized to bEnd.3 cells with 100 ng/ml LPS treatment for 12 hours. Data were presented as means ± SEMs of three independent experiments. **P < 0.01, ***P < 0.001 versus Control group or 100 ng/ml LPS group. [file 12964_2022_862_MOESM8_ESM.docx]

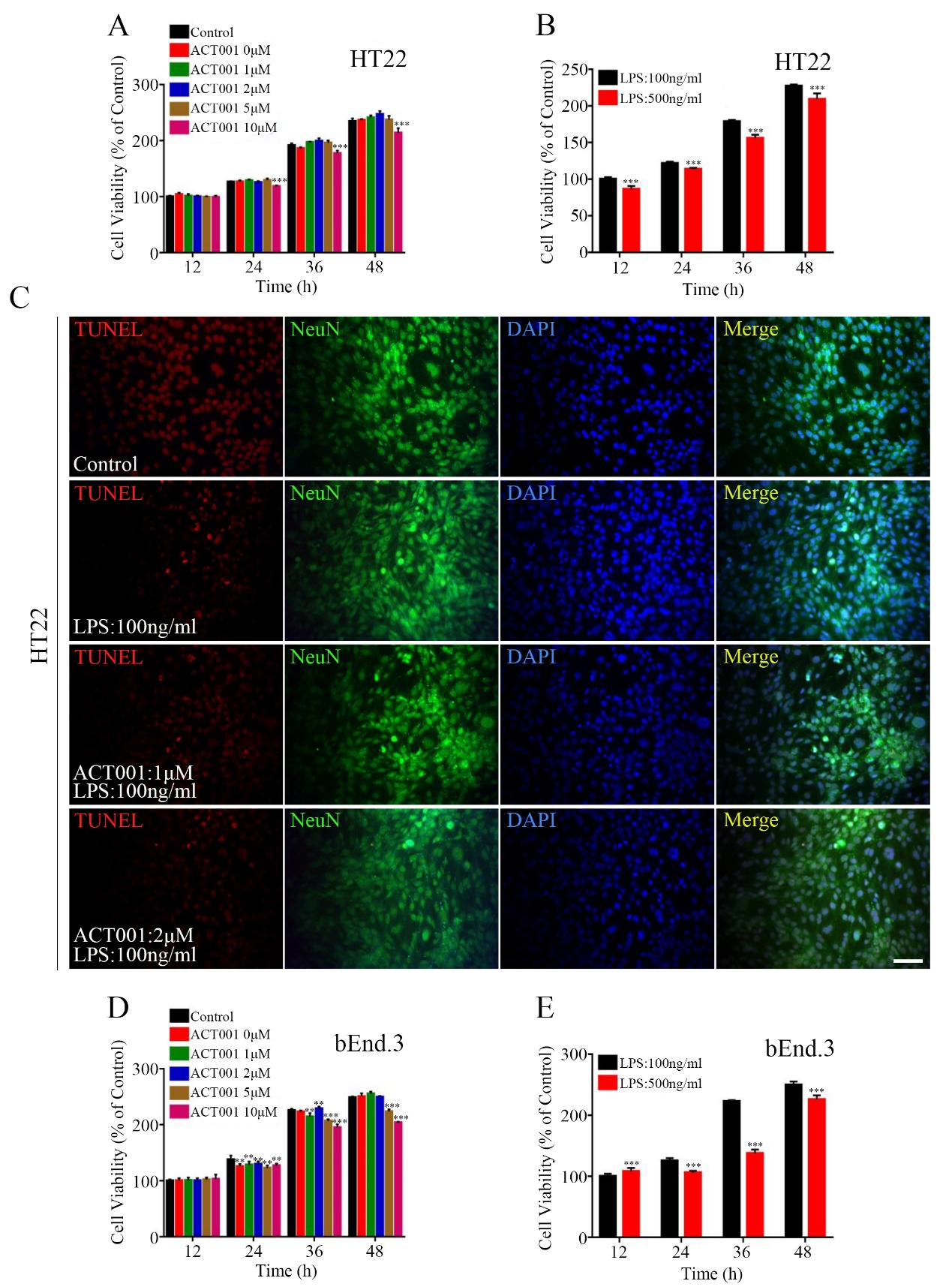


Supplemental Fig. 4 **(A)** HT22 cells were treated with indicated doses of ACT001 for 12-48 hours, then the cytotoxicity of ACT001 was measured by CCK-8 assay. The cell viability result was normalized to HT22 cells without ACT001 treatment (control) for 12 hours. **(B)** HT22 cells were treated with indicated doses of LPS for 12-48 hours, then the cytotoxicity of LPS was measured by CCK-8 assay. The cell viability result was normalized to HT22 cells with 100 ng/ml LPS treatment for 12 hours. **(C)** Representative fluorescence images of NeuN staining with TUNEL labelling in HT22 cells after co-culturing in models for 24 hours. Cell nuclei were shown in blue (DAPI). Scale bar = 100 μm. **(D)** bEnd.3 cells were treated with indicated doses of ACT001 for 12-48 hours, then the cytotoxicity of ACT001 was measured by CCK-8 assay. The cell viability result was normalized to bEnd.3 cells without ACT001 treatment (control) for 12 hours. **(E)** bEnd.3 cells were treated with indicated doses of LPS for 12-48 hours, then the cytotoxicity of LPS was measured by CCK-8 assay. The cell viability result was normalized to bEnd.3 cells with 100 ng/ml LPS treatment for 12 hours. Data were presented as means ± SEMs of three independent experiments. ***P* < 0.01, ****P* < 0.001 vs. control group or 100 ng/ml LPS group.
