# Supplementary material for: ACT001 attenuates microglia-mediated neuroinflammation after traumatic brain injury via inhibiting AKT/NFκB/NLRP3 pathway
Source: Cell Commun Signal. 2022 Apr 23;20:56. doi: 10.1186/s12964-022-00862-y (PMC9035258; doi:10.1186/s12964-022-00862-y)
Supplement: Supplementary file 9 — Additional file 8: Fig. S5 (A-B) Representative fluorescence images for dual staining of NFkB and Iba1 in mouse (A) and rat (B) primary microglia cells after co-treatment with indicated doses of ACT001 and 100 ng/ml LPS for 24 hours. Cell nuclei were shown in blue (DAPI). Scale bar = 10 μm. Cells without ACT001 and LPS treatment were shown as control. [file 12964_2022_862_MOESM9_ESM.docx]

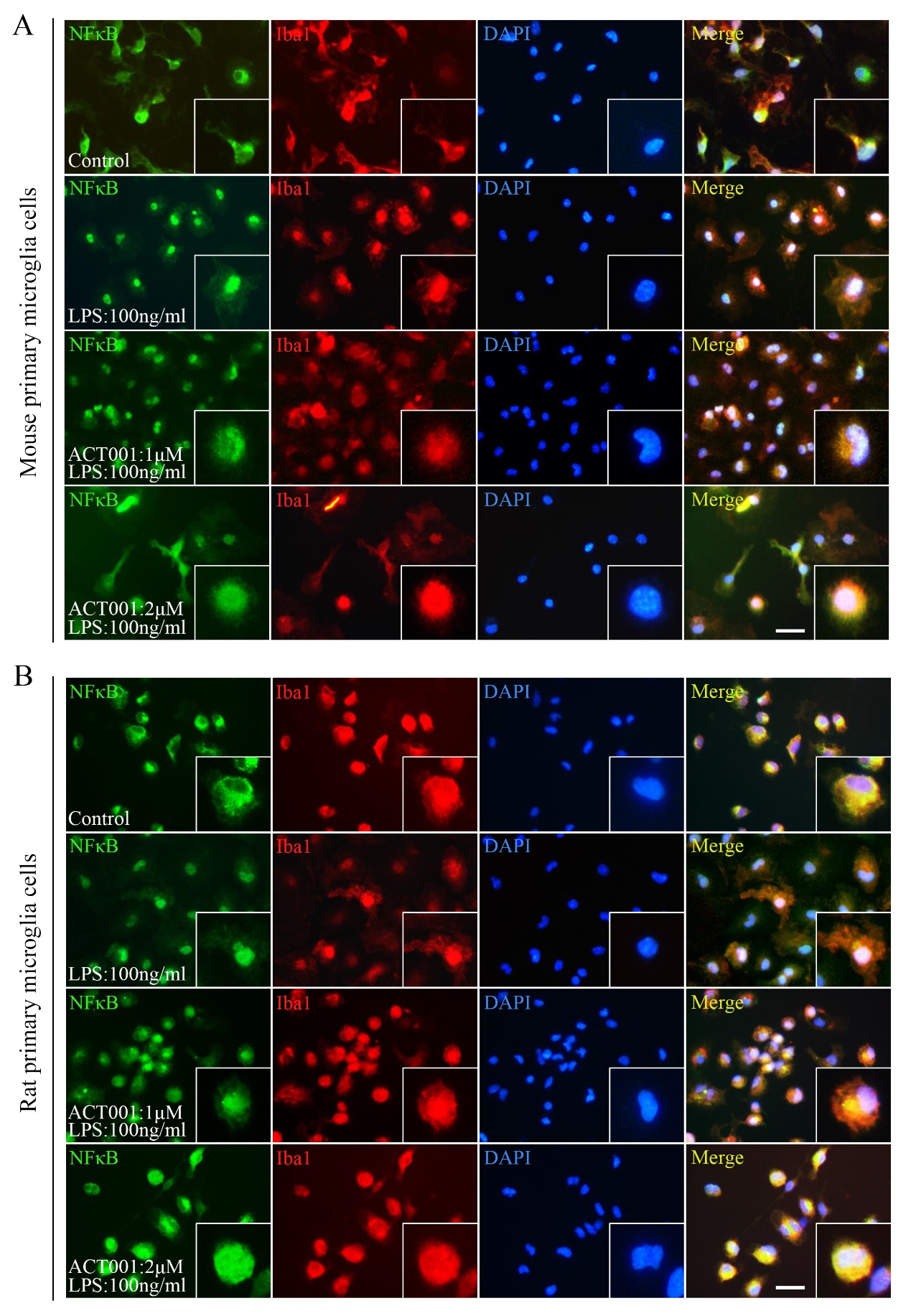


Supplemental Fig. 5 **(A-B)** Representative fluorescence images for dual staining of NFkB and Iba1 in mouse (A) and rat (B) primary microglia cells after co-treatment with indicated doses of ACT001 and 100 ng/ml LPS for 24 hours. Cell nuclei were shown in blue (DAPI). Scale bar = 10 μm. Cells without ACT001 and LPS treatment were shown as control.
